# Dose-response relationship of cadmium and pancreatic cancer risk: a meta-analysis

**DOI:** 10.1136/oemed-2025-110163

**Published:** 2025-09-17

**Authors:** Fu-Jen Lee, Hathaichon Inchai, Jaw-Town Lin, David Koh, Ro-Ting Lin

**Affiliations:** 1 Department of Internal Medicine, Fu Jen Catholic University Hospital, New Taipei City, Taiwan; 2 Graduate Institute of Public Health, College of Public Health, China Medical University, Taichung City, Taiwan; 3 Department of Epidemiology, Faculty of Public Health, Mahidol University, Bangkok, Thailand; 4 Department of Internal Medicine, E-Da Hospital, Kaohsiung City, Taiwan; 5 Saw Swee Hock School of Public Health, National University of Singapore, Singapore; 6 Department of Occupational Safety and Health, College of Public Health, China Medical University, Taichung City, Taiwan

**Keywords:** Cadmium, Meta-analysis, Public health, Gastroenterology, Occupational Health

## Abstract

Cadmium (Cd), a group 1 carcinogen, is linked to the development of pancreatic cancer. Not well-defined is the dose-response relationship between Cd and cancer development.

This study investigated the relationship between Cd exposure and the risk of pancreatic cancer through a meta-analysis focusing on pooled relative risk (RR), biomarker comparisons and dose-response relationships.

This meta-analysis adhered to PRISMA (Preferred Reporting Items for Systematic Reviews and Meta-Analyses) guidelines and applied the PECO (population, exposure, comparator and outcomes) framework. Systematic searches of key databases, including PubMed, Cochrane Library, Web of Science, EMBASE, ScienceDirect and ProQuest, were carried out without language restrictions. Studies that met the predefined PECO criteria were chosen, extracting data on effect estimates such as HRs, ORs, RRs and Cd biomarker concentrations. Analyses involved pooled RRs, standardised mean differences for biomarker comparisons and dose-response relationships, using random-effects meta-analysis and meta-regression. Statistical procedures were performed on R V.4.3.0.

Cd exposure was significantly associated with an increased risk of pancreatic cancer, with a pooled RR of 1.42 (95% CI=1.16 to 1.73). Subgroup analyses confirmed this association in both occupationally and non-occupationally exposed individuals. Dose-response analyses further revealed that higher Cd biomarker levels correlated with greater pancreatic cancer risk (lnRR coefficient=0.610, p=0.035).

Cd exposure is associated with an increased risk of pancreatic cancer, with a dose-response relationship and elevated biomarker levels in cases. Cd exposure is a modifiable risk factor for pancreatic cancer, meriting further investigation.

**WHAT IS ALREADY KNOWN ON THIS TOPIC**

- Cadmium (Cd) exposure may increase pancreatic cancer risks.
- There is limited evidence regarding the dose-response association between Cd exposure and pancreatic cancer risk.

**WHAT THIS STUDY ADDS**

- Cd exposure increased pancreatic cancer risk in both occupationally and non-occupationally exposed individuals.
- Dose-response of Cd exposure and pancreatic cancer was observed.

**HOW THIS STUDY MIGHT AFFECT RESEARCH, PRACTICE OR POLICY**

- Routine biomonitoring of Cd levels can serve as a valuable tool to assess and track the risk of pancreatic cancer, enabling early detection and targeted intervention.

## Introduction

 Pancreatic cancer is one of the most lethal malignancies globally, with low survival and high mortality rates.^1^ Identifying modifiable risk factors earlier is the key to alleviating their impact on public health.^2^ Among the chemicals linked to pancreatic cancer, cadmium (Cd) is a particularly concerning carcinogen because of its widespread environmental and occupational presence, for example, in metalworking, battery production, dietary intake and tobacco smoke.^3 4^ Mechanistically, Cd induces carcinogenesis via oxidative stress, DNA damage and disruption of cellular homeostasis, accumulating in the pancreas over time.^5^

The US Occupational Safety and Health Administration regulations have set biological action levels at 5 µg/L for blood Cd and 3 µg/g creatinine for urinary Cd.^6^ However, typical biomarker levels in the general population are much lower, with median blood Cd levels of 0.40 µg/L and urinary levels of 0.27 µg/g creatinine in the USA.^7^ Recent studies have raised concerns that cadmium exposure—even at concentrations below regulatory action levels—may pose health risks, with recent reviews suggesting subthreshold levels could increase pancreatic cancer risk.^8^ Despite studies comparing Cd biomarker levels across different exposure strata,^9 10^ the dose-response relationship with pancreatic cancer risk at lower levels remains insufficiently characterised.

We reviewed previous meta-analyses investigating the relationship between Cd exposure and pancreatic cancer risk, identifying two studies focused on occupational exposure^11 12^ and two on non-occupational exposure.^13 14^ Meta-analyses of occupational exposure conducted before 2000 yielded inconsistent findings. Ojajärvi *et al*^11^ reported no significant association (meta-risk ratio = 0.70),^11^ while Schwartz and Reis observed a marginally significant positive association (pooled standardised mortality ratio (SMR)=166).^12^ Surprisingly, these studies, conducted in an era of less stringent workplace regulations and potentially higher exposure levels, did not reveal a consistent or elevated risk.

Conversely, more recent studies on non-occupational exposure conducted after the year 2000 indicate a stronger association between cadmium exposure and pancreatic cancer risk, with relative risks (RRs) ranging from 1.62 to 2.05.^13 14^ These later studies benefit from accumulated data and improved diagnostic techniques and included diverse populations, such as those in Asia, beyond the Western occupational cohorts of earlier research.^13 14^ Overall, this newer evidence supports the link between Cd exposure and pancreatic cancer risk, particularly in non-occupational settings.

While existing studies suggest a link between Cd exposure and pancreatic cancer, significant knowledge gaps persist, especially pertaining to the dose-response relationship. This meta-analysis was designed to thoroughly examine the association between Cd exposure and pancreatic cancer risk. We aimed to (1) quantify the association by analysing pooled RR, (2) compare Cd biomarker levels between patients with pancreatic cancer and controls, and (3) investigate the dose-response relationship of cumulative Cd exposure to pancreatic cancer risk.

## Materials and methods

### Review framework

Our meta-analysis conformed to the Preferred Reporting Items for Systematic Reviews and Meta-Analyses (PRISMA) guidelines^15^ (online supplemental material section 1). We applied the population, exposure, comparator and outcomes (PECO) framework to answer the following question: ‘In the general population, what is the relationship between dose-response of cadmium exposure and the risk of pancreatic cancer?’.^16^

### Search strategy

We employed a comprehensive search strategy to identify relevant studies across electronic databases, such as PubMed, Cochrane Library, Web of Science, EMBASE, ScienceDirect and ProQuest. The search used a combination of keywords related to Cd exposure and pancreatic cancer, refined using Boolean operators (AND, OR). The search included all types of literature and imposed no language restrictions, using translation software to include non-English studies. Online supplemental table S1 in online supplemental material section 2 provides the search terms and initial study retrieval counts for pancreatic cancer and Cd exposure in each database.

### Selection of studies and eligibility criteria

Identified studies were imported into Endnote V.21 (Clarivate Analytics, London, UK), and duplicates were removed using Endnote’s Find Duplicate function. Irrelevant studies were excluded after rapid keyword searches and title screening using EndNote. Potential studies were exported to DistillerSR (Evidence Partners, Ottawa, Canada). After removing duplicates identified in DistillerSR, two researchers conducted rapid screening. Studies were selected by screening their titles and abstracts, followed by a full-text review. The eligibility criteria for studies were defined using the PECO framework as follows: (1) population (P), general population; (2) exposure (E), individuals exposed to Cd; (3) comparators (C), individuals without Cd exposure; (4) outcome (O), pancreatic cancer incidence and mortality and (5) studies that either reported effect estimates or provided sufficient information to calculate these estimates.

### Data extraction

Qualitative and quantitative data, extracted from the included studies and entered into Microsoft Excel files, included the following: (1) first author’s name, (2) publication year, (3) country, (4) study period, (5) study design, (6) number and sex of participants, (7) exposure assessment, (8) outcome assessment and (9) declaration of conflicts of interest.

We collected studies that reported outcome measures associated with Cd exposure, including the HR, OR, RR, standardised incidence ratio (SIR) and SMR. The original effect sizes were used for each study. If a study provided the SE of an estimate, it was extracted directly. For studies reporting only 95% CIs, we calculated SE using the following formula:


$$SE=\frac{\ln\left(UpperBound\right)-ln(LowerBound)}{2\times 1.96}$$

where the UpperBound and LowerBound represent the upper and lower bounds of the 95% CIs that were reported in the original study, respectively.

To analyse the differences in Cd biomarker concentrations between patients with pancreatic cancer and the reference population, we extracted the mean biomarker Cd concentrations, SD and sample sizes for both groups. As the biomarker units varied across studies, we standardised the data before calculating the effect sizes where necessary. Hedge’s g was computed to quantify the magnitude of group differences, with a correction factor applied to adjust for small sample bias. The pooled SD and all relevant formulas used in these calculations are detailed in online supplemental material section 3.

### Bias assessment

We conducted a risk of bias assessment following the Office of Health Assessment and Translation (OHAT) framework,^17^ which evaluates seven domains including confounding and outcome assessment. Each study was rated and classified into one of three tiers based on its risk of bias, informing the interpretation of results (online supplemental material section 4 for full criteria and ratings).

### Statistical analyses

Summary effect estimates of the RR and corresponding 95% CIs for Cd exposure and pancreatic cancer were calculated using a random-effects model.^18^ The restricted maximum likelihood (REML) method was employed to estimate the between-study variance, providing an unbiased and efficient measure of heterogeneity, particularly in the presence of high variability among studies.^18^ To enhance the robustness of the random-effects CIs, the Hartung-Knapp adjustment was applied.^19^ Heterogeneity across studies was evaluated using the *I*^2^ test and Cochran’s Q test.^18^ The tau-squared (τ^2^) value, derived from the REML method, was used to quantify between-study variances.^18^ Potential publication bias was assessed using Egger’s test and visualised using a funnel plot.^18^ To address potential small-study effects identified by Egger’s test, we conducted a sensitivity analysis comparing fixed-effect and random-effects models, as recommended by the Cochrane Handbook.^18^ Since random-effects models give relatively more weight to smaller studies, substantial differences between the two approaches can indicate the influence of small-study effects on the pooled estimate. Subgroup analyses were conducted to evaluate pooled effects stratified by region and exposure status.

We performed a meta-regression analysis to examine the dose-response relationship between Cd exposure and the RR of pancreatic cancer. The regression analysis used the mean Cd exposure level as the independent variable and natural log-transformed RR (lnRR) as the dependent variable, with the standard errors of lnRR incorporated as weights to account for the precision of the estimates. A sensitivity analysis was conducted to assess the consistency of the pooled RR estimates by comparing two different meta-analysis models. Given the lack of a universally established threshold for identifying influential studies, we applied two predefined criteria to evaluate the robustness of our findings. The main model included all eligible studies, while the alternative model sequentially excluded studies based on the following approaches. (1) Studies at a high risk of bias (tier 2 studies, classified based on online supplemental material section 4) were excluded, which assessed whether lower-quality studies influenced the pooled estimate. (2) Top 10% wt criterion: the top 10% of the study subgroups with the highest weights were excluded to evaluate the influence of dominant studies on the pooled RR estimate. The effect of these exclusions on the pooled RR estimates and heterogeneity (*I*^2^) was examined to determine the sensitivity of the conclusions. By systematically removing potentially influential studies, this stepwise approach provides a structured assessment of the robustness of the meta-analysis results. To address potential bias arising from statistical dependency due to multiple effect estimates within the same study, we conducted a sensitivity analysis using only one effect estimate per study. When studies reported multiple exposure levels (eg, low, medium, high), we retained the medium level; when only two levels were reported (eg, high and medium), we again retained the medium estimate. This approach reduces the risk of dependency bias and ensures greater independence of effect estimates for publication bias assessment, while maintaining representativeness of the evidence. All analyses were performed in R, V.4.3.0 (R Core Team, Vienna, Austria), using the ‘meta’ and ‘metafor’ packages for meta-analysis and associated tools for graphical and diagnostic assessments. After completing the analyses, we assessed the certainty of the evidence, using the Grading of Recommendations, Assessment, Development and Evaluations (GRADE) approach,^20^ considering factors such as risk of bias, inconsistency, imprecision and potential for publication bias. Summary assessments are presented in online supplemental material section 5.

## Results

### Study selection and characteristics

**Figure 1:**
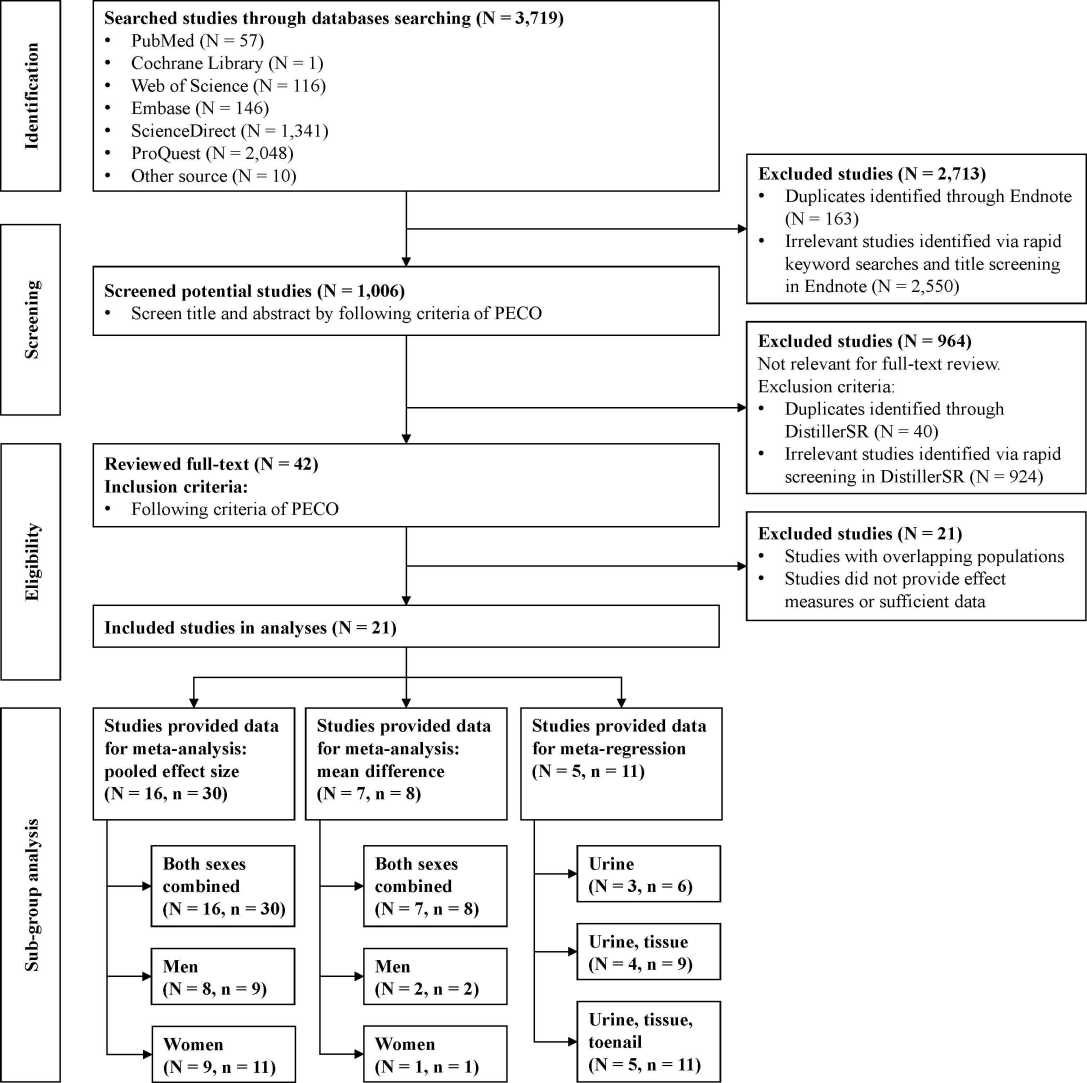
Flowchart of study selection. N, number of studies; n, number of study groups; PECO, population, exposure, comparator and outcomes.

We identified 3719 studies using five databases (figure 1). After excluding 163 duplicate and 2550 irrelevant studies, 1006 potential studies were assessed based on the PECO criteria. Of these, 964 were excluded after title and abstract screening because they did not meet the criteria for full-text review. Specifically, 40 duplicates were identified using DistillerSR, and 924 were deemed irrelevant. Finally, 42 studies were included in this review. During this phase, 21 studies were excluded owing to overlapping populations or insufficient effect measures and data. Ultimately, 21 studies were included in the analysis. Of these, 16 studies (number of study groups (n) = 30) provided data for the meta-regression of pooled effect size,^910 21^,^34^ 7 studies (n=8) supplied data for the meta-analysis,^1022 35^,^39^ and 5 studies (n=11) reported data for the meta-regression of Cd biomarkers.^9 21 24 25 27^ Online supplemental material section 6 summarises the characteristics of the included studies (online supplemental table S2), the risk-of-bias assessment for individual studies (online supplemental table S3), and presents additional results (online supplemental figure S1-S14 and table S4) as described in the following sections.

### Evaluation of the relationship between exposure to cadmium and the risk of pancreatic cancer

**Figure 2:**
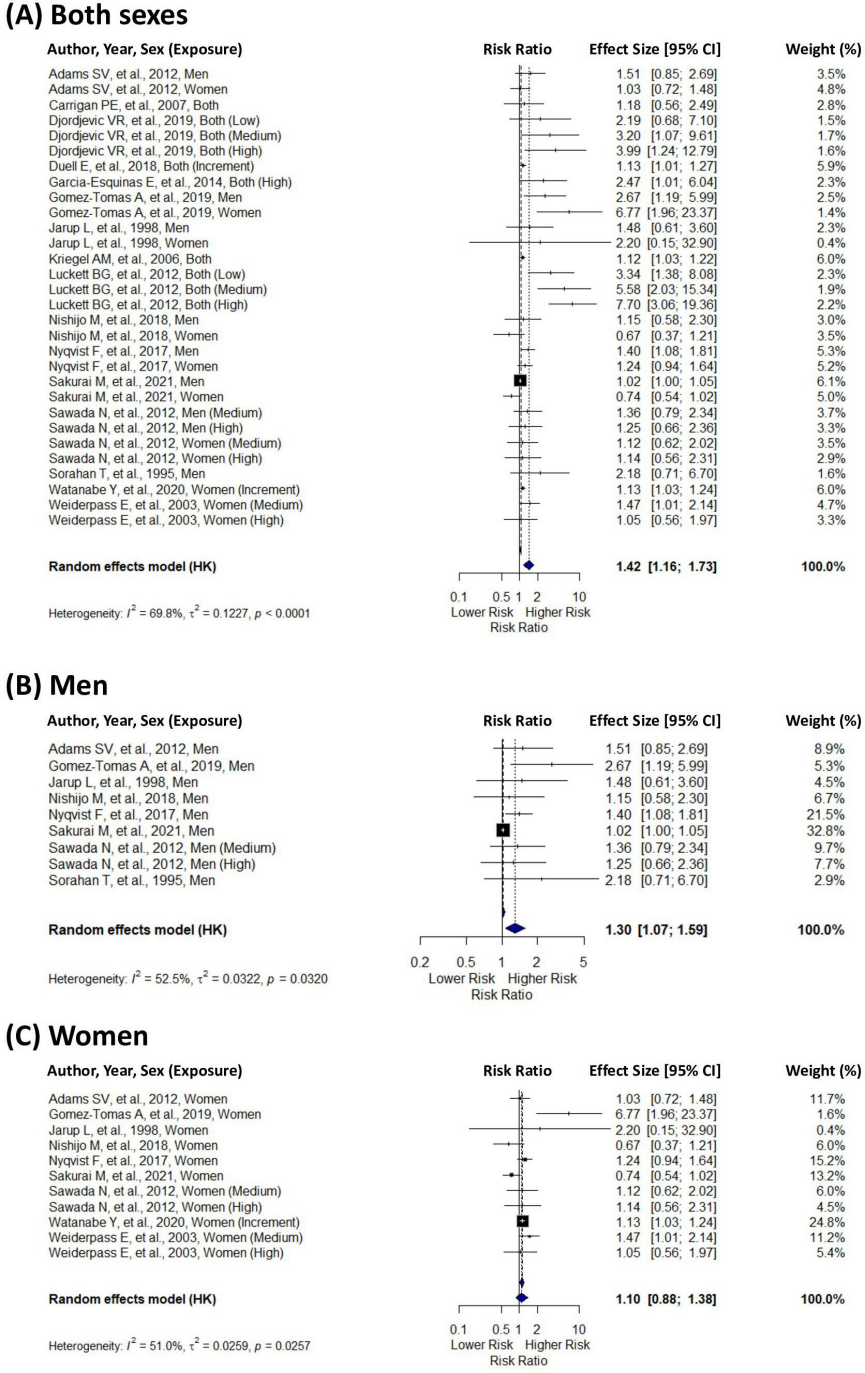
Forest plot of the pooled effect of cadmium exposure on pancreatic cancer risk, by sex.

We analysed the pooled effect sizes from multiple measures (HR, OR, RR, SIR and SMR) and calculated the RR for different sexes. A significant association was found between Cd exposure and the risk of pancreatic cancer. For both sexes combined, the pooled RR was 1.42 (95% CI=1.16 to 1.73), suggesting increased pancreatic cancer risk associated with Cd exposure (figure 2). For men, the pooled RR was 1.30 (95% CI=1.07 to 1.59), indicating increased risk. In contrast, for women, the pooled RR was 1.10 (95% CI=0.88 to 1.38), showing a less pronounced and statistically non-significant increase in risk.

We conducted a subgroup analysis to assess the robustness of the pooled RR. By region, we observed a significant association between Cd exposure and all regions, with the highest risk in North America (pooled RR=2.33, 95% CI=1.14 to 4.76), followed by Europe (pooled RR=1.51, 95% CI=1.17 to 1.95) (online supplemental figure S1). By exposure status, we observed that the pool RRs of pancreatic cancer were significant and of similar magnitude in both non-workers (pooled RR=1.46, 95% CI=1.15 to 1.86) and workers (pooled RR=1.41, 95% CI=1.09 to 1.83) (online supplemental figure S2).

In addition to RRs, the study examined the standardised mean differences (SMDs) (using Hedge’s *g*) between the groups. For both sexes combined, the SMD was g = 1.26 (95% CI=−0.33 to 2.85), indicating a higher but not statistically significant Cd concentration in the case group compared with the control group (online supplemental figure S3). Among men, the result was higher with g = 3.79 (95% CI=−21.84 to 29.43), reflecting greater variability in the difference, which may require further investigation to determine its clinical significance.

**Figure 3:**
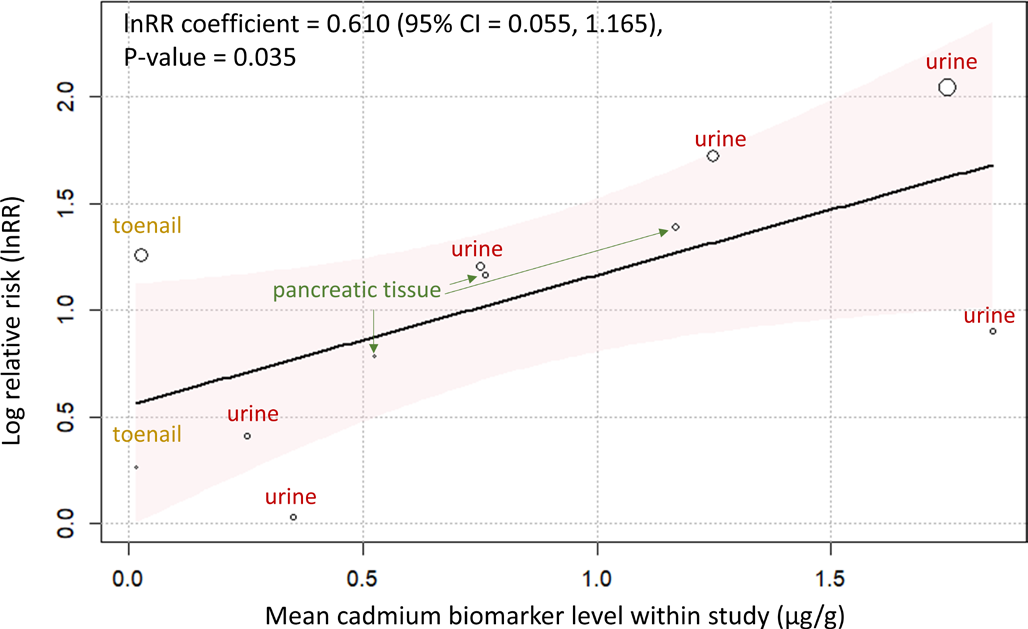
Meta-regression of the association between cadmium (Cd) exposure and pancreatic cancer risk. The plot shows the dose-response relationship between cadmium concentration (μg/g) and the natural log-transformed relative risk (lnRR) for pancreatic cancer, based on a random-effects meta-regression model. The solid line represents the fitted trend, with the shaded area indicating the 95% CI. Each point represents a study-specific group, defined by biomarker type (urine, pancreatic tissue or toenail). Cadmium concentrations reflect values reported within individual studies, and the lnRR represents the estimated change in risk per 1 µg/g increase in exposure. Note: Some elevated lnRR values at near-zero exposure levels reflect comparisons to reference groups with extremely low background Cd levels, which may exaggerate relative differences even when absolute exposures are low.

Meta-regression analysis of various Cd biomarkers revealed significant positive associations between Cd exposure and risk of pancreatic cancer. Mixed biomarkers, including urine, pancreatic tissue and toenails, showed a lnRR coefficient of 0.610 (95% CI=0.055 to 1.165, p=0.035) (figure 3). Specifically, urinary Cd alone had the highest but non-significant regression coefficient of 0.808 (95% CI=−0.272 to 1.887, p=0.106), while the combination of urinary and pancreatic tissue Cd demonstrated the second highest coefficient of 0.786 (95% CI=0.105 to 1.467) and the lowest p value (p=0.029) (online supplemental figure S4). These results indicate that although individual biomarkers may vary in their strength of association, all biomarkers consistently demonstrated a positive link between Cd accumulation and pancreatic cancer risk, with an overall significant pooled effect.

Our sensitivity analysis revealed a 2.8% decrease in pooled RRs and a 3.9% increase in *I*^2^ between the main and alternative models when studies classified as tier 2 were excluded (online supplemental figure S5A). After excluding the top 10% of the highest-weighted studies in the main model, we observed an 8.5% increase in pooled RRs and a 9.6% decrease in *I*^2^ between the main and alternative models (online supplemental figure S5B). Egger’s test for publication bias indicated potential small-study effects (bias coefficient=1.38, 95% CI=0.84 to 1.92, p<0.001) (online supplemental fiigure S6). Given the evidence of publication bias, we compared fixed-effects and random-effects models to assess the influence of small-study effects. The fixed-effect model yielded a substantially lower pooled estimate compared with the random-effects model, suggesting that smaller studies may be driving the effect towards a more positive effect, indicating potential small-study effects that warrant further scrutiny (online supplemental figure S7). An additional sensitivity analysis using only one effect estimate per study to address potential statistical dependency yielded consistent results with our main findings (online supplemental figure S8-S14). To assess whether funnel plot asymmetry was influenced by exposure-level-dependent variance patterns, publication bias analysis using only one effect estimate per study showed similar results. Egger’s test bias coefficient was 1.26 (95% CI=0.65 to 1.88, p=0.0006), confirming that the observed small-study effects persisted regardless of the analytical approach. This meta-analysis examined the association between Cd exposure and pancreatic cancer risk with the following certainty of evidence: (1) pooled RR analysis—low certainty; (2) Cd biomarker comparison—very low certainty; and (3) dose-response analysis—low certainty (online supplemental table S4).

## Discussion

The study confirmed a significant association between Cd exposure and increased pancreatic cancer risk, in both occupationally and non-occupationally exposed individuals. Biomarker analysis indicated higher Cd levels in patients than in controls, although the increase was not statistically significant. Meta-regression analysis showed a significant dose-response relationship between Cd biomarkers and cancer risk, particularly when combining urinary and tissue levels.

Compared with previous meta-analyses, which often focused on specific populations or included fewer studies,^11^,^14^ our meta-analysis offers a more comprehensive evaluation by incorporating both occupational and non-occupational populations and integrating diverse methods, including pooled RR, SMD and meta-regression analyses (table 1). Our approach confirmed a significant pooled RR and also uniquely highlighted higher Cd biomarker levels in patients with pancreatic cancer and the presence of a dose-response relationship. Further analysis demonstrated a clear dose-response relationship across different biomarkers, indicating that monitoring Cd exposure and assessing pancreatic cancer risk could have broader and more versatile applications.

**Table 1:** Comparison of our meta-analysis with previous meta-analyses

Main population	Study	Included studies (publication year)	Main findings	Reference
Workers and the general population	Our meta-analysis	16 studies with 30 study groups for meta-analysis 7 studies with 8 study groups for standardised mean difference analysis 5 studies with 11 study groups for meta-regression analysis	Significant positive association: Pooled RR=1.42 (95% CI=1.16 to 1.73) for both sexes combined Higher pooled RR in men than in women Elevated cadmium biomarker levels in patients with pancreatic cancer compared with controls Significant dose-response relationship between cadmium biomarker levels and pancreatic cancer risk (p=0.035)	–
Workers	Ojajärvi *et al* (2000)	2 cohort studies with 2 study groups in Sweden (1985)	Non-significant association: Meta-risk ratio=0.7 (95% CI=0.4 to 1.4)	^11^
Workers	Schwartz *et al* (2000)	3 cohort studies with 3 study groups: 2 in Sweden (1985, 1998) and 1 in the UK (1995)	Marginally significant positive association: Pooled SMR=166 (95% CI=98 to 280)	^12^
General population	Chen *et al* (2015)	4 cohort studies with 4 study groups: 2 in Japan (2011, 2012) and 2 in the USA (2012, 2014) 2 case-control studies with two study groups: 1 in Spain (2012) and one in the USA (2012)	Significant positive association: Pooled RR=2.05 (95% CI=1.58 to 2.66) Higher pooled RR in men (RR=1.78, 95% CI=1.04 to 3.05) than in women (RR=1.02, 95% CI=0.63 to 1.65)	^13^
General population	Farahmandian *et al* (2023)	2 cohort studies with two study groups: 1 in Japan (2012) and one in Spain (2020) 6 case-control studies with six study groups: 1 in Egypt (2006), 1 in Serbia (2019), 2 in Spain (2012, 2019) and 2 in the USA (2007, 2012)	Significant positive association: Pooled RR=1.62 (95% CI=1.18 to 2.21)	^14^

RR, relative risk; SMR, standardised mortality ratio.

Dose-response analyses showed that higher Cd biomarker levels were associated with increased pancreatic cancer risk. Our work is the first meta-analysis on Cd biomarkers and pancreatic cancer risk. Significant positive correlations were found with urine Cd alone or in combination with other biomarkers, such as pancreatic tissue and toenails. Beyond pancreatic cancer, Cd exposure has been extensively studied for its potential link to other cancers, including breast, lung and prostate cancers.^40^,^43^ Notably, for breast cancer, findings are inconsistent: a 2015 meta-analysis reported a significant association, with an OR of 2.24, indicating high Cd exposure may increase risk,^40^ whereas a 2020 study found no significant association when dietary or urinary Cd was assessed, particularly in post-menopausal women.^41^ Furthermore, lung cancer consistently showed a strong positive association, with a pooled RR of 1.68 for increased urinary Cd levels.^42^ However, prostate cancer studies revealed no significant link, with ORs for increasing exposure quartiles ranging from 1.03 to 1.16, indicating Cd exposure may not contribute meaningfully to risk.^43^ These findings highlight the varying degrees of association depending on the cancer type and exposure metrics. Importantly, our meta-regression analysis underscores the utility of diverse Cd biomarkers, including urine, pancreatic tissue and toenail Cd levels, for assessing pancreatic cancer risk. Mixed biomarkers demonstrated a significant positive association, with the combination of urinary and pancreatic tissue Cd showing the strongest link, surpassing the association observed when toenail Cd was included. This finding suggests that urinary and pancreatic tissue Cd biomarkers offer complementary strengths in exposure assessment: urinary Cd reflects cumulative systemic exposure, whereas pancreatic tissue Cd captures organ-specific accumulation,^5^ ^9 44^ even at concentrations below occupational action levels. Future research should harmonise protocols and leverage advanced analytical and artificial intelligence technologies to improve biomarker reliability and enhance our understanding of the role of Cd in pancreatic cancer.^45^

Further, we observed a stronger association in men than in women, which may reflect differences in Cd exposure patterns, biological susceptibility and lifestyle factors. Men are more likely to experience higher occupational exposure and cancer burden,^46^ ^47^ particularly in industries such as metalworking and battery production. Smoking, a major source of Cd exposure, is also more prevalent among men.^48^ Biological differences, such as variations in Cd metabolism and detoxification, may contribute further. Studies have suggested that oestrogen has a protective effect against the development of pancreatic cancer,^49^ with β-oestradiol shown to mitigate Cd toxicity in pancreatic islet cells.^50^ However, most cohort studies have not accounted for menopausal status or exogenous hormone use, potentially confounding the observed differences.^21^ ^24^ ^51^ Additionally, compared with men, women tend to consume more antioxidant-rich fruits and vegetables, which may help mitigate Cd-induced oxidative stress and lower its carcinogenic potential.^52^,^54^ This combination of sex-based exposure patterns, biological mechanisms and lifestyle factors highlights the multifactorial nature of pancreatic cancer.

Our sensitivity analyses showed that the pooled RR was affected by study selection and weighting, highlighting the influence of study characteristics on meta-analysis outcomes. Excluding tier 2 studies led to a lower pooled RR and higher *I*^2^, indicating that the inclusion of tier 2 studies may have contributed to an overestimation of the association and masked part of the underlying heterogeneity. Removing the top 10% of high-weight studies resulted in a higher pooled RR and lower *I*^2^, suggesting that the inclusion of these larger, more precise studies may have lessened the effect estimate and increased heterogeneity. This bidirectional shift implies that true association likely falls between these estimates, emphasising the need for careful evaluation of study quality, weighting and the impact of smaller studies in meta-analytical models.

The study has several limitations. First, the moderate heterogeneity and few dose-response analyses indicate variability in study designs, populations and exposure assessments, affecting the robustness and generalisability of the findings. Second, not all studies adequately adjusted for key confounders such as smoking, other chemical exposures and dietary habits, which may have overestimated the pancreatic cancer risk from Cd exposure. Third, Egger’s test suggested potential small-study effects, where smaller studies with positive findings might have disproportionately influenced the pooled results, possibly inflating the observed association. However, we addressed potential statistical dependency from multiple effect estimates per study through sensitivity analysis, which demonstrated consistent results and confirmed that our conclusions were not driven by within-study correlation of estimates. The persistence of funnel plot asymmetry in sensitivity analysis using one effect estimate per study confirms that genuine small-study effects, rather than exposure-level-dependent variance patterns, are driving the observed publication bias. These issues highlight the need for larger, well-designed studies to confirm this association and reduce publication bias and methodological inconsistencies.

This study has several strengths. First, adherence to the PRISMA guidelines and the inclusion of diverse study designs (cohort, case-control, occupational and non-occupational) ensured methodological rigour. Second, as the first meta-analysis to examine the dose-response relationship using multiple biomarkers, including urinary Cd, pancreatic tissue and toenails, it offers fresh evidence of the role of Cd in cancer risk. Third, the use of a large dataset, long follow-up periods and random-effects models may have enhanced the reliability of our findings.

In conclusion, this study showed that higher levels of Cd exposure were associated with increased pancreatic cancer risk. The significant dose-response relationship underscores the need for enhanced environmental monitoring and targeted interventions for high-risk groups, including smokers and workers in Cd-intensive industries. While exposure levels varied across studies, the Cd biomarker concentrations included in our analyses were generally below occupational action levels, suggesting that risk from Cd exposure may still be detectable at environmentally relevant concentrations in the general population.

## Supplementary material

10.1136/oemed-2025-110163 online supplemental file 1

## Data Availability

Data are available upon reasonable request.
