# Supplementary material for: Dose-response relationship of cadmium and pancreatic cancer risk: a meta-analysis
Source: Occup Environ Med. 2025 Sep 17;82(8):e110163. doi: 10.1136/oemed-2025-110163 (PMC12573421; doi:10.1136/oemed-2025-110163)
Supplement: online supplemental file 1 [file oemed-82-8-s001.pdf]

Supplementary File

Table of Content

Supplementary Material Section 1 3

PRISMA 2020 Checklist 3

Supplementary Material Section 2 5

Table S1. Search terms and initial study retrieval counts for pancreatic cancer and cadmium exposure in each database 5

Supplementary Material Section 3 6

Supplementary Material Section 4 7

Risk of Bias Assessment 7

Tier Classification Criteria 7

Supplementary Material Section 5 8

Certainty of Evidence 8

Supplementary Material Section 6 9

Table S2. Characteristics of studies included in the meta-regression and meta-analysis 9

Table S3. Summary of risk of bias of human epidemiological studies using the OHAT risk of bias rating tool for pancreatic cancer 16

Figure S1. Subgroup analysis between cadmium exposure and pancreatic risk, by regions 17

Figure S2. Subgroup analysis between cadmium exposure and pancreatic risk, by exposure status 18

Figure S3. Standardized mean difference in cadmium levels between pancreatic cancer patients and the reference group, by sex 19

Figure S4. Meta-regression of the association between cadmium exposure and pancreatic cancer risk. 20

Figure S5. Sensitivity analyses of pooled relative risk for pancreatic cancer by alternative models: (a) by excluding studies at high risk of bias, categorized as Tier 2, (b) by excluding studies with top 10% of the highest weights in the main model. 21

Figure S6. Funnel plot for the risk ratio from the articles included in the meta-analysis 22

Figure S7. Forest plots comparing fixed-effect and random-effects estimates........................ 23

Figure S8. Forest plot of the pooled effect of cadmium exposure on pancreatic cancer risk, by sex (sensitivity analysis using one effect estimate per study) 24

Figure S9. Meta-regression of the association between cadmium exposure and pancreatic cancer risk (sensitivity analysis using one effect estimate per study) 25

Figure S10. Subgroup analysis between cadmium exposure and pancreatic cancer risk, by regions (sensitivity analysis using one effect estimate per study)... 26

Figure S11. Subgroup analysis between cadmium exposure and pancreatic cancer risk, by exposure status (sensitivity analysis using one effect estimate per study) 27

Figure S12. Meta-regression of the association between cadmium exposure and pancreatic cancer risk (sensitivity analysis using one effect estimate per study). 28

Figure S13. Comparison of main analysis and sensitivity analysis using one effect estimate per study: (a) by excluding studies at high risk of bias, categorized as Tier 2, (b) by excluding studies with top 10% of the highest weights in the main model.. 29

Figure S14. Funnel plot for the risk ratio from the articles included in the meta-analysis (sensitivity analysis using one effect estimate per study)... 30

Table S4. Summary of the certainty of evidence 31

References...32

Supplementary Material Section 1

PRISMA 2020 Checklist

Section and Topic	Item #	Checklist item	Location where item is reported
**TITLE**			
Title	1	Identify the report as a systematic review.	P.1 (Title page) Identify as a meta-analysis
**ABSTRACT**			
Abstract	2	See the PRISMA 2020 for Abstracts checklist.	P.2 (Abstract page)
**INTRODUCTION**			
Rationale	3	Describe the rationale for the review in the context of existing knowledge.	P.5–6
Objectives	4	Provide an explicit statement of the objective(s) or question(s) the review addresses.	P.5–6
**METHODS**			
Eligibility criteria	5	Specify the inclusion and exclusion criteria for the review and how studies were grouped for the syntheses.	P.7–8
Information sources	6	Specify all databases, registers, websites, organisations, reference lists and other sources searched or consulted to identify studies. Specify the date when each source was last searched or consulted.	P.7, Supplementary Material Section 2
Search strategy	7	Present the full search strategies for all databases, registers and websites, including any filters and limits used.	Supplementary Material Section 2
Selection process	8	Specify the methods used to decide whether a study met the inclusion criteria of the review, including how many reviewers screened each record and each report retrieved, whether they worked independently, and if applicable, details of automation tools used in the process.	P.7–8
Data collection process	9	Specify the methods used to collect data from reports, including how many reviewers collected data from each report, whether they worked independently, any processes for obtaining or confirming data from study investigators, and if applicable, details of automation tools used in the process.	P.7–8
Data items	10a	List and define all outcomes for which data were sought. Specify whether all results that were compatible with each outcome domain in each study were sought (e.g. for all measures, time points, analyses), and if not, the methods used to decide which results to collect.	P.8–9
	10b	List and define all other variables for which data were sought (e.g. participant and intervention characteristics, funding sources). Describe any assumptions made about any missing or unclear information.	P.8
Study risk of bias assessment	11	Specify the methods used to assess risk of bias in the included studies, including details of the tool(s) used, how many reviewers assessed each study and whether they worked independently, and if applicable, details of automation tools used in the process.	P.9, Supplementary Material Section 4
Effect measures	12	Specify for each outcome the effect measure(s) (e.g. risk ratio, mean difference) used in the synthesis or presentation of results.	P.8–9, Supplementary Material Section 3
Synthesis methods	13a	Describe the processes used to decide which studies were eligible for each synthesis (e.g. tabulating the study intervention characteristics and comparing against the planned groups for each synthesis (item #5)).	P.8–9, Supplementary Material Section 3
	13b	Describe any methods required to prepare the data for presentation or synthesis, such as handling of missing summary statistics, or data conversions.	P.8–9, Supplementary Material Section 3
	13c	Describe any methods used to tabulate or visually display results of individual studies and syntheses.	P.8–9, Supplementary Material Section 3
	13d	Describe any methods used to synthesize results and provide a rationale for the choice(s). If meta-analysis was performed, describe the model(s), method(s) to identify the presence and extent of statistical heterogeneity, and software package(s) used.	P.8–1
	13e	Describe any methods used to explore possible causes of heterogeneity among study results (e.g. subgroup analysis, meta-regression).	P.9–11
	13f	Describe any sensitivity analyses conducted to assess robustness of the synthesized results.	P.10–11

Section and Topic	Item #	Checklist item	Location where item is reported
Reporting bias assessment	14	Describe any methods used to assess risk of bias due to missing results in a synthesis (arising from reporting biases).	P.9, Supplementary Material Section 4
Certainty assessment	15	Describe any methods used to assess certainty (or confidence) in the body of evidence for an outcome.	P.11, Supplementary Material Section 5
**RESULTS**			
Study selection	16a	Describe the results of the search and selection process, from the number of records identified in the search to the number of studies included in the review, ideally using a flow diagram.	P.12
	16b	Cite studies that might appear to meet the inclusion criteria, but which were excluded, and explain why they were excluded.	P.12
Study characteristics	17	Cite each included study and present its characteristics.	P.12 Supplementary Material Section 6: Table S2
Risk of bias in studies	18	Present assessments of risk of bias for each included study.	Supplementary Material Section 6: Table S3
Results of individual studies	19	For all outcomes, present, for each study: (a) summary statistics for each group (where appropriate) and (b) an effect estimate and its precision (e.g. confidence/credible interval), ideally using structured tables or plots.	Supplementary Material Section 6: Table S2
Results of syntheses	20a	For each synthesis, briefly summarise the characteristics and risk of bias among contributing studies.	Supplementary Material Section 6: Table S3
	20b	Present results of all statistical syntheses conducted. If meta-analysis was done, present for each the summary estimate and its precision (e.g. confidence/credible interval) and measures of statistical heterogeneity. If comparing groups, describe the direction of the effect.	P.12–14
	20c	Present results of all investigations of possible causes of heterogeneity among study results.	Figure 2, Supplementary Material Section 6: Figures S1–S4
	20d	Present results of all sensitivity analyses conducted to assess the robustness of the synthesized results.	P.14, Supplementary Material Section 6: Figure S5, S8–S14
Reporting biases	21	Present assessments of risk of bias due to missing results (arising from reporting biases) for each synthesis assessed.	Supplementary Material Section 6: Figures S7–S14
Certainty of evidence	22	Present assessments of certainty (or confidence) in the body of evidence for each outcome assessed.	P.14, Supplementary Material Section 6: Table S4
**DISCUSSION**			
Discussion	23a	Provide a general interpretation of the results in the context of other evidence.	P.15
	23b	Discuss any limitations of the evidence included in the review.	P.18–19
	23c	Discuss any limitations of the review processes used.	P.18–19
	23d	Discuss implications of the results for practice, policy, and future research.	P.15–20
**OTHER INFORMATION**			
Registration and protocol	24a	Provide registration information for the review, including register name and registration number, or state that the review was not registered.	The review was not registered
	24b	Indicate where the review protocol can be accessed, or state that a protocol was not prepared.	A protocol was not prepared
	24c	Describe and explain any amendments to information provided at registration or in the protocol.	Not reported
Support	25	Describe sources of financial or non-financial support for the review, and the role of the funders or sponsors in the review.	P.21
Competing interests	26	Declare any competing interests of review authors.	P.21
Availability of data, code and other materials	27	Report which of the following are publicly available and where they can be found: template data collection forms; data extracted from included studies; data used for all analyses; analytic code; any other materials used in the review.	Not reported

Supplementary Material Section 2

We developed the search terms for our outcome of interest, pancreatic cancer, based on MeSH terminology. Specifically, we used (“pancreatic neoplasm” OR “pancreatic neoplasms” OR “pancreatic cancer” OR “pancreatic ductal adenocarcinoma” OR “PDAC”) to retrieve relevant literature on pancreatic cancer. For the exposure variable, cadmium, we used “cadmium” as the primary search term. Combined, our study’s search string was (“pancreatic neoplasm” OR “pancreatic neoplasms” OR “pancreatic cancer” OR “pancreatic ductal adenocarcinoma” OR “PDAC”) AND (“cadmium”). Below, the table displays the search terms used and the initial number of studies retrieved. Each source was last searched on May 23, 2024.

Table S1. Search terms and initial study retrieval counts for pancreatic cancer and cadmium exposure in each database

Database	Search term	Counts
PubMed	(“pancreatic neoplasm” OR “pancreatic neoplasms” OR “pancreatic cancer” OR “pancreatic ductal adenocarcinoma” OR “PDAC”) AND (“cadmium”)	57
Cochrane Library	(“pancreatic neoplasm” OR “pancreatic neoplasms” OR “pancreatic cancer” OR “pancreatic ductal adenocarcinoma” OR “PDAC”) AND (“cadmium”)	1
Web of Science	(“pancreatic neoplasm” OR “pancreatic neoplasms” OR “pancreatic cancer” OR “pancreatic ductal adenocarcinoma” OR “PDAC”) AND (“cadmium”)	116
Embase	(“pancreatic neoplasm” OR “pancreatic neoplasms” OR “pancreatic cancer” OR “pancreatic ductal adenocarcinoma” OR “PDAC”) AND (“cadmium”)	146
ScienceDirect	(“pancreatic neoplasm” OR “pancreatic neoplasms” OR “pancreatic cancer” OR “pancreatic ductal adenocarcinoma” OR “PDAC”) AND (“cadmium”)	1,341
ProQuest	(“pancreatic neoplasm” OR “pancreatic neoplasms” OR “pancreatic cancer” OR “pancreatic ductal adenocarcinoma” OR “PDAC”) AND (“cadmium”)	2,048

Supplementary Material Section 3

We calculated Cohen's d and then applied a correction factor to account for small sample size bias, converting it to Hedge's g. Hedge's g was computed as follows [2]:

$$g = d \times J$$

where Cohen's d was calculated to quantify the magnitude of the difference between two groups using the formula [2]:

$$d = \frac{Mean_{pancreatic\ cancer} - Mean_{reference}}{SD_{pooled}}$$

The pooled standard deviation (SD_{pooled}) is defined as [2]:

$$SD_{pooled} = \sqrt{\frac{(n_1 - 1) \times {SD_1}^2 + (n_2 - 1) \times {SD_2}^2}{df}}$$

where df is the number of degrees of freedom, typically $n_1 + n_2 - 2$. Moreover, n_1 and n_2 represent the sample sizes, and SD_1 and SD_2 indicate the standard deviations of the pancreatic cancer and reference groups, respectively. J is the correction factor for small sample bias, calculated as [2]:

$$J = 1 - \frac{3}{4(df) - 1}$$

Supplementary Material Section 4

Risk of Bias Assessment

To evaluate the quality and reliability of individual studies, we conducted a risk of bias assessment following the Office of Health Assessment and Translation (OHAT) framework [1]. Each study was categorized into tiers 1–3, reflecting its overall risk of bias, which directly informed our conclusions.

The OHAT risk of bias tool evaluates studies across seven domains, including confounding, detection, and selection biases. Each study was rated as having a definitely low, probably low, probably high, or definitely high risk of bias. If a study lacked sufficient information to assess specific domains confidently, it was classified as having a probably high risk of bias.

Two independent researchers (FJL and RTL) conducted the assessments separately. In cases of disagreement, they engaged in discussions to reach a consensus.

The OHAT risk of bias tier system places particular emphasis on the following key elements:

- Does the study design or analysis appropriately account for key confounding variables?
- Can we be confident in the characterization of exposure?
- Can we be confident in the outcome assessment?

Tier Classification Criteria

Each study was assigned to one of the following three tiers based on its risk of bias:

- Tier 1: Studies with a "definitely low" or "probably low" risk of bias in key domains, along with most other applicable items rated as low risk.
- Tier 2: Studies that do not meet the Tier 1 or Tier 3 criteria, indicating moderate confidence in their reliability.
- Tier 3: Studies rated as having a "definitely high" or "probably high" risk of bias in key domains, along with most other applicable items classified as high risk.

This tiered approach allows for a structured integration of risk of bias considerations across studies, ensuring a more comprehensive evaluation of health outcomes.

Supplementary Material Section 5

Certainty of Evidence

The Grading of Recommendations, Assessment, Development, and Evaluations (GRADE) approach provides a systematic framework for assessing the certainty of evidence in determining the true association between exposure and outcome [3]. Evidence is categorized into four levels: “high,” “moderate,” “low,” or “very low” certainty.

By default, observational studies are initially rated as “low to moderate” certainty evidence [1]. However, the certainty rating may be downgraded due to concerns related to:

- Risk of bias (e.g., methodological limitations in individual studies),
- Inconsistency (e.g., unexplained heterogeneity across studies),
- Indirectness (e.g., differences between study populations or exposures and the research question),
- Imprecision (e.g., wide confidence intervals, small sample sizes), or
- Publication bias (e.g., selective reporting, industry-funded studies).

Conversely, the certainty of evidence may be upgraded when:

- A large effect size is observed,
- A clear dose-response relationship exists, or
- Plausible confounding factors suggest the true effect might be even stronger than observed.

A summary of findings regarding the certainty of evidence is presented in **Table S4** in **Supplementary Material Section 6**.

Supplementary Material Section 6

Table S2. Characteristics of studies included in the meta-regression and meta-analysis

First author's name [Reference]	Publication year	Country	Study period	Study design	Number of participants	Exposure assessment	Outcome assessment	Note
Adams SV, et al. [4]	2012	United States of America	1988–2006	Cohort	Follow-up:<br>• Both sexes: 15,675<br>• Men: 7,455<br>• Women: 8,218<br><br>Case:<br>• Both sexes: 37<br>• Men: 23<br>• Women: 14	Urinary Cd (µg/g creatinine)<br><br>Cut points for men:<br>• Q1: 0.153<br>• Q2: 0.297<br>• Q3: 0.580<br>• Geometric mean: 0.252<br><br>Cut points for women:<br>• Q1: 0.210<br>• Q2: 0.418<br>• Q3: 0.819<br>• Geometric mean: 0.352	Adjusted HR per 2-fold uCd increase:<br>• Men: 1.51 (95% CI = 0.85–2.70)<br>• Women: 1.03 (95% CI = 0.72–1.48)	A 2-fold increase in uCd corresponds approximately to quartile boundaries.
Amaral AFS, et al. [5]	2012	Spain	1992–2001	Case-control	Case:<br>• Both sexes: 118<br><br>Control:<br>• Both sexes: 399	Toenail Cd (µg/g)<br><br>Mean ± SE:<br>• Case: 0.08 ± 0.02<br>• Control: 0.10 ± 0.04	Adjusted OR:<br>• Low vs. reference: 0.87 (95% CI = 0.37–2.03)<br>• Medium vs. reference: 2.04 (95% CI = 1.00–4.17)<br>• High vs. reference: 3.58 (95% CI = 1.86–6.88)	The study population overlaps with that of Gomez-Tomas A et al.'s study, which was published later in 2019. Therefore, we used the estimated OR from Gomez-Tomas A et al. We used the mean difference of Cd between cases and controls in this study for the standardized mean difference calculation.
Baralic K, et al. [6]	2022	Serbia	2019–2021	Case-control	Case:<br>• Men: 12	Blood Cd (µg/L)<br><br>Percentiles: Case vs. control:	No relevant estimates.	For the 25th-50th-75th quantiles, the mean is estimated as:

First author's name [Reference]	Publication year	Country	Study period	Study design	Number of participants	Exposure assessment	Outcome assessment	Note
					Control:<br>• Men: 66	• 25th: 19.35 vs. 0.7858<br>• 50th: 20.95 vs. 1.102<br>• 75th: 31.30 vs. 1.542		$\text{Mean} \approx \frac{Q_{25} + Q_{50} + Q_{75}}{3}$<br>The SD is estimated based on Wan et al. (2014) [7].
Carrigan PE, et al. [8]	2007	United States of America	2003–2004	Case-control	Case:<br>• Both sexes: 35<br><br>Control:<br>• Both sexes: 35	Pancreatic juice Cd (µg/L)<br>Mean ± SD:<br>• Case: 1.2 ± 1.3<br>• Control: 1.1 ± 1.1	Adjusted OR, per 1.1 µg/L pancreatic juice Cd increase: 1.18 (95% CI = 0.56–2.50)	The original paper reported a value of 1.56 for lower confidence interval. However, after reviewing other meal concentrations in the same Table, the corrected value should be 0.56.
Djordjevic VR, et al. [9]	2019	Serbia	2014–2016	Case-control	Case:<br>• Both sexes: 31<br><br>Control:<br>• Both sexes: 29	Pancreatic tissue Cd (µg/g)<br><br>Cut points:<br>• Reference: < 0.491<br>• Second quartile (low): 0.491–0.558<br>• Middle quartile (medium): 0.558–0.966<br>• Upper quartile (high): ≥ 0.966	OR:<br>• Second (low) vs. reference: 2.193 (95% CI = 0.677–7.100)<br>• Middle (medium) vs. reference: 3.200 (95% CI = 1.051–9.473)<br>• Upper (high) vs. reference: 3.990 (95% CI = 1.136–11.679)	
Duell E, et al. [10]	2018	Europe	Not reported (Median follow-up: 12.2 years)	Case-control	Case:<br>• Men: 429<br><br>Control:<br>• Men: 902	Blood Cd (µg/L)	Adjusted OR:<br>• Per log2-transformed Cd increase: 1.13 (95% CI = 1.01–1.27)<br>• Q5 vs. Q1: 1.87 (95% CI = 1.13–3.08)	The OR for per log2-transformed Cd increase was selected over the Q5 vs. Q1 as it includes more participants, ensuring better representation of the overall population.

First author's name [Reference]	Publication year	Country	Study period	Study design	Number of participants	Exposure assessment	Outcome assessment	Note
Farzin L, et al. [11]	2013	Iran	2010–2011	Case-control	Case:<br>• Both sexes: 80<br>• Men: 46<br>• Women: 34<br><br>Control:<br>• Both sexes: 100<br>• Men: 55<br>• Women: 45	Blood Cd (µg/L)<br><br>Mean ± SD for both sexes:<br>• Case: 3.10 ± 1.05<br>• Control: 1.52 ± 0.88<br><br>Mean ± SD for men:<br>• Case: 3.19 ± 0.97<br>• Control: 1.50 ± 0.89<br><br>Mean ± SD for women:<br>• Case: 2.98 ± 1.10<br>• Control: 1.54 ± 0.87	No relevant estimates.	
Forte G, et al. [12]	2024	Italy	Not reported	Case-control	Case:<br>• Both sexes: 46<br><br>Control:<br>• Both sexes: 20	Blood Cd (ng/mL)<br><br>Percentiles: Case vs. control:<br>• 5th: 0.21 vs. 0.11<br>• 50th: 0.58 vs. 0.50<br>• 95th: 1.52 vs. 1.57	No relevant estimates.	For the 5th-50th-95th quantiles, the mean is estimated as:<br>$\text{Mean} \approx \frac{Q_5 + 2 \times Q_{50} + Q_{95}}{4}$<br><br>SD is estimated as:<br>$\text{SD} \approx \frac{Q_{95} - Q_5}{2 \times 1.645}$
Garcia-Esquinas E, et al. [13]	2014	United States of America	1989–2008	Cohort	Follow-up:<br>• Both sexes: 3,792<br><br>Case:<br>• Both sexes: 24	Urinary Cd (µg/g creatinine)<br><br>Cut points:<br>• Low: < 1.23<br>• High: ≥ 1.23<br><br>Percentiles:<br>• 20th: 0.55<br>• 80th: 1.62	Adjusted HR:<br>• High vs. low: 2.47 (95% CI = 1.01–6.03)<br>• 80th vs. 20th: 2.40 (95% CI = 1.39–4.17)	The HR for high vs. low was selected over the 80th vs. 20th percentile as it includes more participants, ensuring better representation of the overall population.

First author's name [Reference]	Publication year	Country	Study period	Study design	Number of participants	Exposure assessment	Outcome assessment	Note
Gomez-Tomas A, et al. [14]	2019	Spain	1992–2001	Case-control	Case:<br>• Both sexes: 78<br>• Men: 47<br>• Women: 31<br><br>Control:<br>• Both sexes: 416	Toenail Cd (μg/g)<br><br>Cut points:<br>• Reference: ≤ 0.01<br>• Medium: 0.01–0.02<br>• High: > 0.02	Adjusted OR for both sexes:<br>• Medium vs. reference: 1.30 (95% CI = 0.67–2.53)<br>• High vs. reference: 3.52 (95% CI = 1.99–6.20)<br><br>Adjusted OR for men:<br>• High vs. reference: 2.67 (95% CI = 1.19–5.98)<br><br>Adjusted OR for women:<br>• High vs. reference: 6.77 (95% CI = 1.96–23.36)	In this meta-analysis for both sexes, if the original paper provides estimates for both sexes, men, and women separately, we prioritize using the combined men and women data. If unavailable, we use the both sexes data, followed by single-sex estimates as a last resort.
Jarup L, et al. [15]	1998	Sweden	1931–1992	Cohort	Follow-up:<br>• Both sexes: 869<br><br>Case:<br>• Both sexes: 7<br>• Men: 6<br>• Women: 1	Battery workers	SMR, compared with Kalmar county:<br>• Men: 148 (95% CI = 54.5–323)<br>• Women: 220 (95% CI = 5.5–1230)	
Kriegel AM, et al.[16]	2006	Egypt	2001–2002	Case-control	Case:<br>• Both sexes: 31<br><br>Control:<br>• Both sexes: 52	Serum Cd (ng/mL)<br><br>Mean ± SD<br>• Case: 11.1 ± 7.7<br>• Control: 7.1 ± 5.0	Adjusted OR: 1.12 (95% CI = 1.04–1.23)	
Luckett BG, et al. [17]	2012	United States of America	2001–2005	Case-control	Case:<br>• Both sexes: 69<br><br>Control:<br>• Both sexes: 158	Urinary Cd (μg/g creatinine)<br><br>Cut points:<br>• Reference: < 0.5<br>• Low: 0.5 to < 1<br>• Medium: 1 to < 1.5<br>• High: ≥ 1.5	Adjusted OR:<br>• Low vs. reference: 3.34 (95% CI = 1.38–8.07)<br>• Medium vs. reference: 5.58 (95% CI = 2.03–15.34)<br>• High vs. reference: 7.70 (95% CI = 3.06–19.34)	

First author's name [Reference]	Publication year	Country	Study period	Study design	Number of participants	Exposure assessment	Outcome assessment	Note
Nishijo M, et al. [18]	2018	Japan	1979–2005	Cohort	Follow-up:<br>Exposed / Reference:<br>• Both sexes: 7,348 / 2,098<br>• Men: 3,363 / 926<br>• Women: 3,985 / 1,172<br><br>Case:<br>Exposed / Reference:<br>• Both sexes: 75 / 23<br>• Men: 41 / 9<br>• Women: 34 / 14	Exposed: Residents lived in the Cd-polluted Jinzu River basin areas<br>• No exposure and borderline<br>• Mild<br>• Moderate<br>• High<br><br>Reference: Non-polluted sections of two towns and five cities	Adjusted HR for men:<br>• Exposed vs. reference: 1.15 (95% CI = 0.6–2.4)<br>• Mild vs. no/borderline: 1.11 (95% CI = 0.53–2.32)<br>• Moderate vs. no/borderline: 1.19 (95% CI = 0.47–3.02)<br>• High vs. no/borderline: 0.54 (95% CI = 0.17–1.67)<br><br>Adjusted HR for women:<br>• Exposed vs. reference: 0.67 (95% CI = 0.4–1.3)<br>• Mild vs. no/borderline: 0.88 (95% CI = 0.39–2.01)<br>• Moderate vs. no/borderline: 0.59 (95% CI = 0.17–2.13)<br>• High vs. no/borderline: 1.10 (95% CI = 0.40–2.99)	The HR for exposed vs. reference was selected over the other comparisons as it includes more participants, ensuring better representation of the overall population.
Nyqvist F, et al. [19]	2017	Sweden	1979–2010	Cohort	Follow-up:<br>• Both sexes: 34,254<br><br>Case:<br>• Both sexes: 114<br>• Men: 61<br>• Women: 53	Exposed: Residents lived within a 2-km radius from 12 contaminated glassworks sites<br><br>Reference:<br>• County<br>• National	SIR, compared with county<br>• Men: 1.35 (95% CI = 1.04–1.74)<br>• Women: 1.18 (95% CI = 0.88–1.54)<br><br>SIR, comparing with national<br>• Men: 1.40 (95% CI = 1.07–1.79)<br>• Women: 1.24 (95% CI = 0.93–1.62)	SMR was selected over SIR as it reflects both incidence and survival, providing a more comprehensive measure of disease burden. National-level comparisons were preferred over county-level to ensure greater generalizability, minimize regional biases, and use more stable reference rates.

First author's name [Reference]	Publication year	Country	Study period	Study design	Number of participants	Exposure assessment	Outcome assessment	Note
Sakurai M, et al. [20]	2021	Japan	1981–2016	Cohort	Follow-up:<br>• Both sexes: 3,139<br>• Men: 1,404<br>• Women: 1,735<br><br>Case:<br>• Both sexes: 63<br>• Men: 30<br>• Women: 33	Urinary β$_2$-microglobulin (β$_2$-MG) concentration (μg/g creatinine), a marker of renal tubular dysfunction resulting from Cd exposure	Adjusted RR, per 1,000 β$_2$-MG increase:<br>• Men: 1.02 (95% CI = 1.00–1.05)<br>• Women: 0.74 (95% CI = 0.54–1.03)	
Sawada N, et al. [21]	2012	Japan	1990–2006	Cohort	Follow-up:<br>• Both sexes: 90,383<br>• Men: 42,032<br>• Women: 48,351<br><br>Case:<br>• Both sexes: 236<br>• Men: 123<br>• Women: 113	Dietary Cd intake (μg/day)<br><br>Men, median intake<br>• Low: 19.7<br>• Middle: 26.7<br>• High: 35.4<br><br>Women<br>• Low: 19.2<br>• Middle: 24.9<br>• High: 32.3	Adjusted HR for men:<br>• Middle vs. low: 1.36 (95% CI = 0.79–2.34)<br>• High vs. low: 1.25 (95% CI = 0.66–2.36)<br><br>Adjusted HR for women:<br>• Middle vs. low: 1.12 (95% CI = 0.62–2.02)<br>• High vs. low: 1.14 (95% CI = 0.56–2.30)	
Sen G. [22]	2015	United Kingdom	Not reported	Case-control	Case:<br>• Both sexes: 25<br><br>Control<br>• Both sexes: 34	Bile Cd (mg/L)<br><br>Mean ± SD:<br>• Case: 0.84864 ± 1.063557<br>• Control: 0.41497 ± 1.002480	No relevant estimates.	
Sorahan T, et al. [23]	1995	United Kingdom	1946–1992	Cohort	Follow-up:<br>• Men: 347<br><br>Case:<br>• Men: 4	Alloy workers in factories A and B	SMR, compared with the general population of England and Wales:<br>• Men: 218 (95% CI = 59–558)	
Watanabe Y, et al. [24]	2020	Japan	1993–2012	Cohort	Follow-up:<br>• Both sexes: 2,804	Urinary Cd (μg/g creatinine)	Adjusted RR, per unit urine Cd increase:	

First author's name [Reference]	Publication year	Country	Study period	Study design	Number of participants	Exposure assessment	Outcome assessment	Note
					• Men: 1,107<br>• Women: 1,697<br><br>Case:<br>• Both sexes: 23<br>• Men: 8<br>• Women: 15		• Men: Not reported<br>• Women: 1.13 (95% CI = 1.03–1.24)	
Weiderpass E, et al. [25]	2003	Finland	1971–1995	Cohort	Follow-up:<br>• Women: 413,877<br><br>Case:<br>• Women: 1,302	Workplace Cd concentration (µg/m^3)<br><br>Cut point between low and high exposure levels: 0.23	Adjusted RR:<br>• Low vs. no exposure: 1.47 (95% CI = 1.01–2.14)<br>• High vs. no exposure: 1.05 (95% CI = 0.56–1.97)	

Abbreviations: Cd, cadmium; CI, confidence interval; HR, hazard ratio; OR: odds ratio; Q: quantile; RR = relative risk or risk ratio; SE = standard error; SIR = standardized incidence ratio; SMR = standardized mortality ratio.

Table S3. Summary of risk of bias of human epidemiological studies using the OHAT risk of bias rating tool for pancreatic cancer

Symbol	Meaning
++	Definitely low risk of bias
+	Probably low risk of bias
-	Probably high risk of bias
--	Definitely high risk of bias

Bias domain	Adams SV, et al. (2012)	Amaral AFS, et al. (2012)	Baralic K, et al. (2022)	Carrigan PE, et al. (2007)	Djordjevic VR, et al. (2019)	Duell E, et al. (2018)	Farzin L, et al. (2013)	Forte G, et al. (2024)	Garcia-Esquinas E, et al. (2014)	Gomez-Tomas A, et al. (2019)	Jarup L, et al. (1998)	Kriegel AM, et al. (2006)	Luckett BG, et al. (2012)	Nishijo M, et al. (2018)	Nyqvist F, et al. (2017)	Sakurai M, et al. (2021)	Sawada N, et al. (2012)	Sen G. (2015)	Sorahan T, et al. (1995)	Watanabe Y, et al. (2020)	Weiderpass E, et al. (2003)
**Confounding bias**																					
1. Did the study design or analysis account for important confounding and modifying variables? **(Key domain)**	+	+	-	+	-	+	--	+	+	+	-	+	+	+	-	+	+	--	-	+	+
**Detection bias**																					
1. Can we be confident in the exposure characterization? **(Key domain)**	++	++	++	++	++	++	++	++	++	++	+	++	++	++	+	+	+	+	+	++	+
2. Can we be confident in the outcome assessment? **(Key domain)**	++	+	+	+	++	+	+	++	++	++	++	++	++	++	++	++	++	++	++	++	++
**Selection bias**																					
1. Did selection of study participants result in appropriate comparison groups?	+	+	+	+	+	+	+	-	+	+	-	+	+	+	-	+	++	-	+	+	+
**Attrition/Exclusion bias**																					
1. Were outcome data complete without attrition or exclusion from analysis?	+	+	-	+	-	-	-	-	++	+	+	++	++	+	+	++	+	-	-	+	++
**Selective reporting bias**																					
1. Were all measured outcome reported?	++	++	+	++	-	+	++	++	++	++	+	++	++	++	+	++	++	+	+	-	+
**Other bias**																					
1. Conflict of interest	+	+	+	-	+	-	-	+	+	+	-	+	-	+	+	+	+	-	-	+	-
**Summary tier category**	T1	T1	T2	T1	T2	T1	T2	T1	T1	T1	T2	T1	T1	T1	T2	T1	T1	T2	T2	T1	T1
**Included in meta-analysis: Pooled effect size**	V			V	V	V			V	V	V	V	V	V	V	V	V		V	V	V
**Included in meta-analysis: Mean difference**		V	V	V			V	V				V						V			
**Included in meta-regression**	V				V				V	V			V								

By region

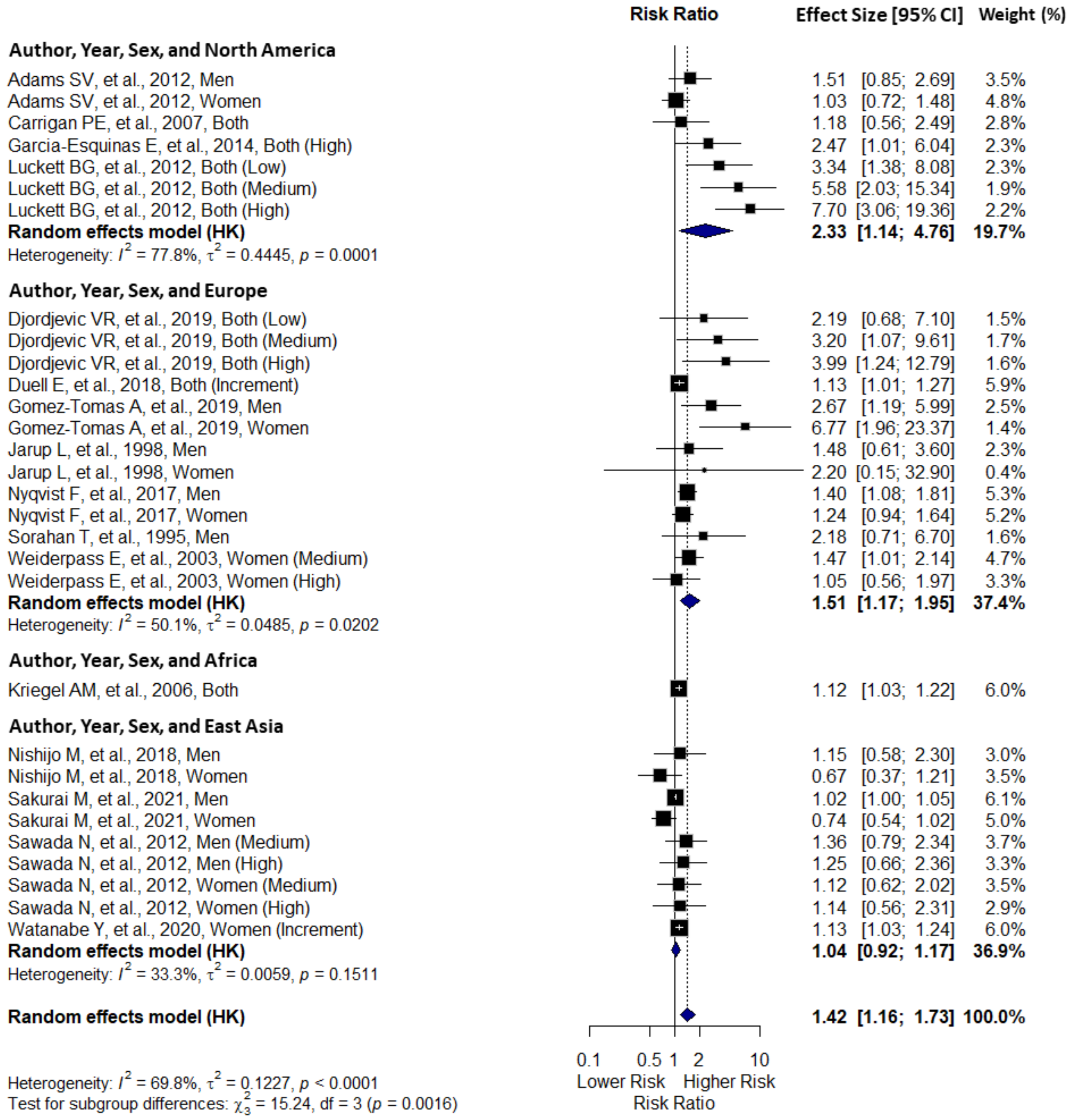

Figure S1. Subgroup analysis between cadmium exposure and pancreatic risk, by regions

By exposure status

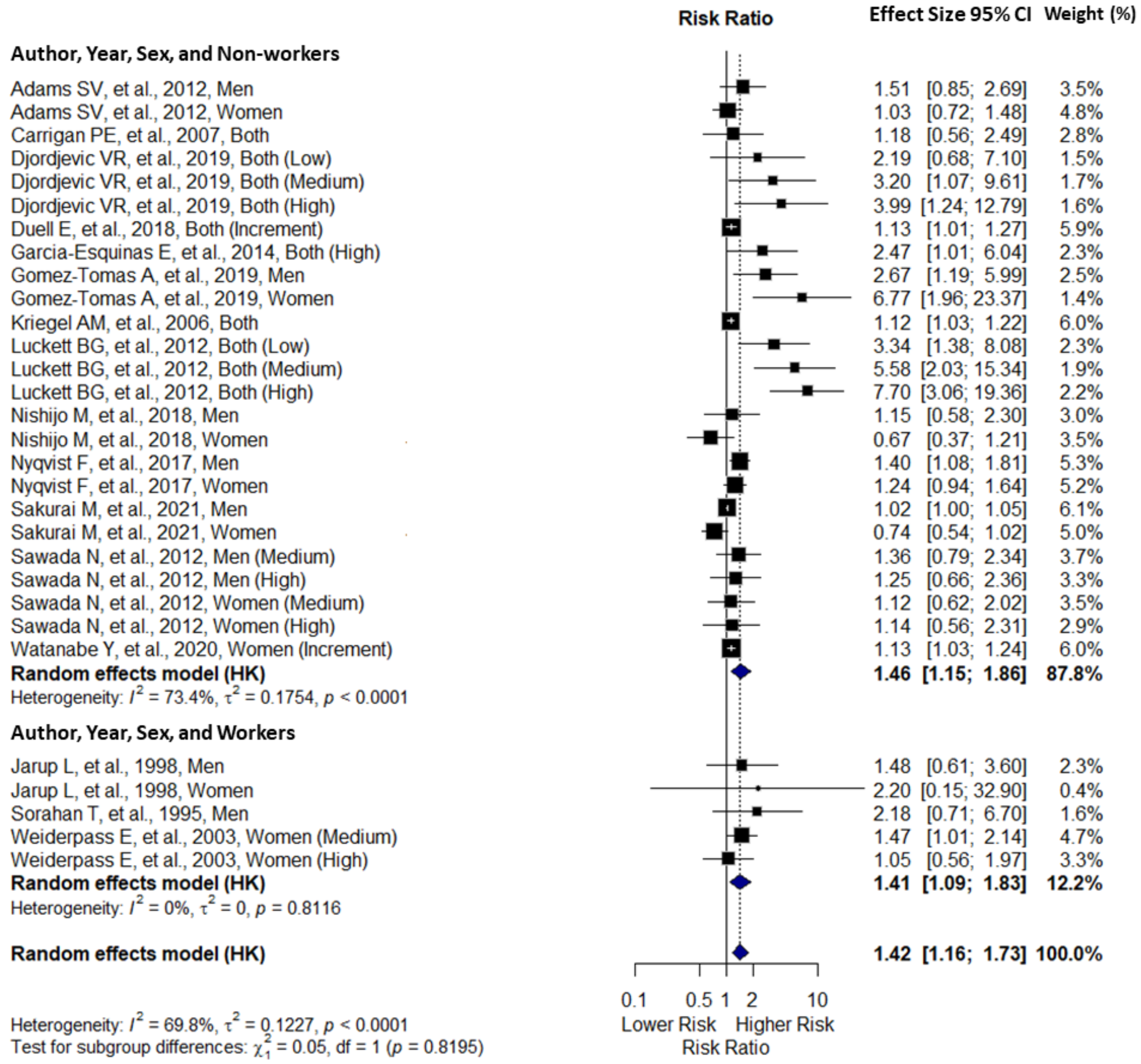

Figure S2. Subgroup analysis between cadmium exposure and pancreatic risk, by exposure status

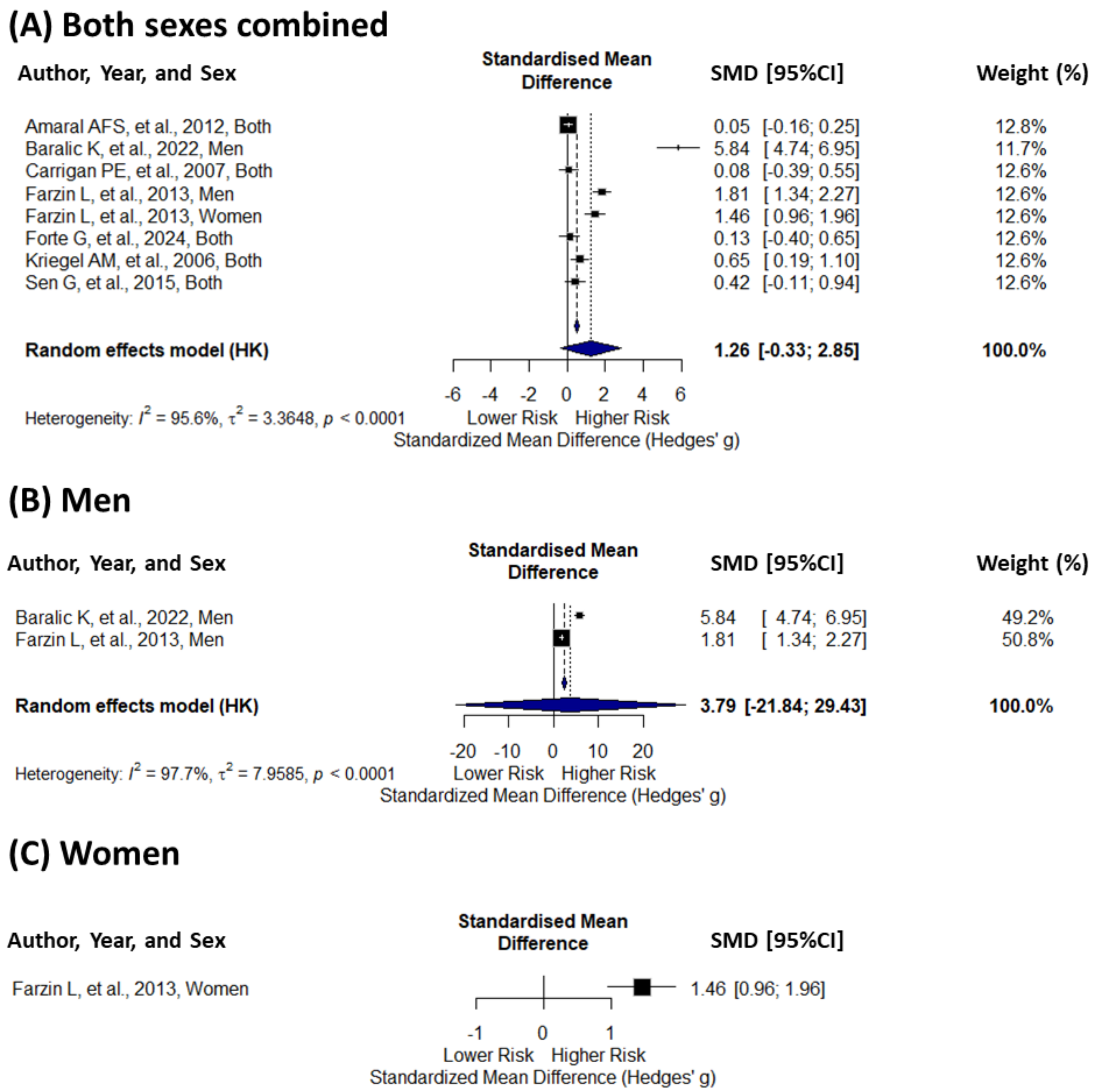

Figure S3. Standardized mean difference in cadmium levels between pancreatic cancer patients and the reference group, by sex

(A) Urine and pancreatic tissue

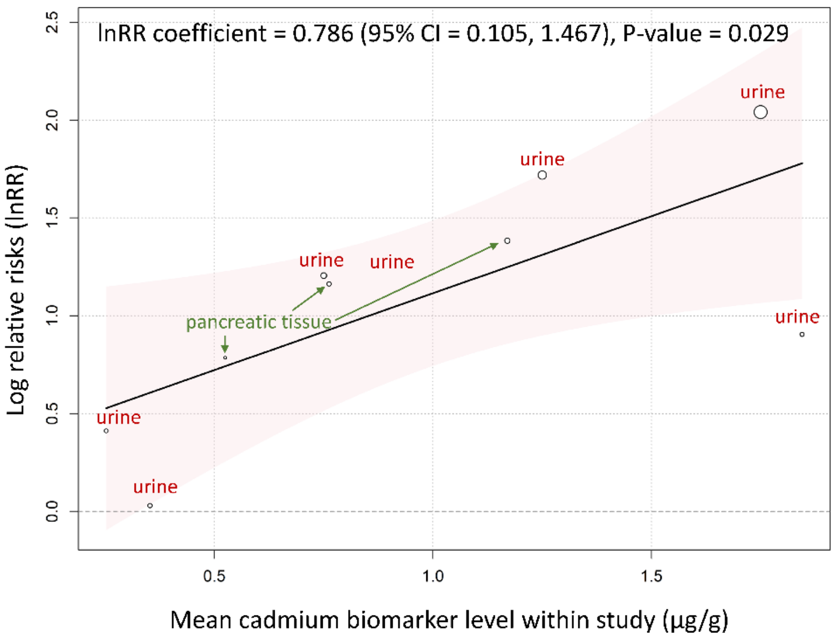

(B) Urine

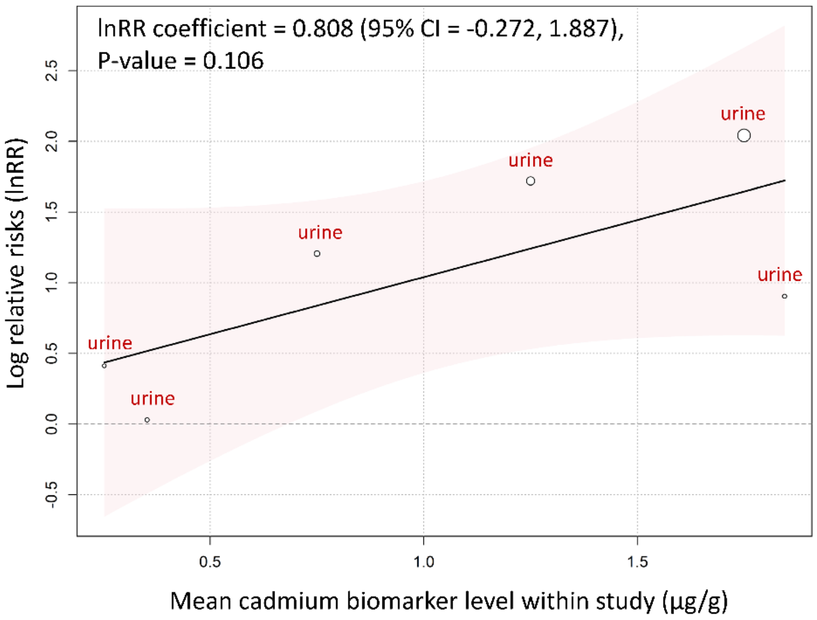

Figure S4. Meta-regression of the association between cadmium exposure and pancreatic cancer risk.

The plot shows the dose-response relationship between cadmium concentration (µg/g) and the natural log-transformed relative risk (lnRR) for pancreatic cancer, based on a random-effects meta-regression model. The solid line represents the fitted trend, and the shaded area indicates the 95% confidence interval (CI). Each point represents a study-specific group, defined by biomarker type: (A) urine and pancreatic tissue; (B) urine only. Cadmium concentrations reflect values reported within individual studies, and the lnRR represents the estimated change in risk per 1 µg/g increase in exposure.

(A) Excluding studies at high risk of bias (Tier 2 studies)

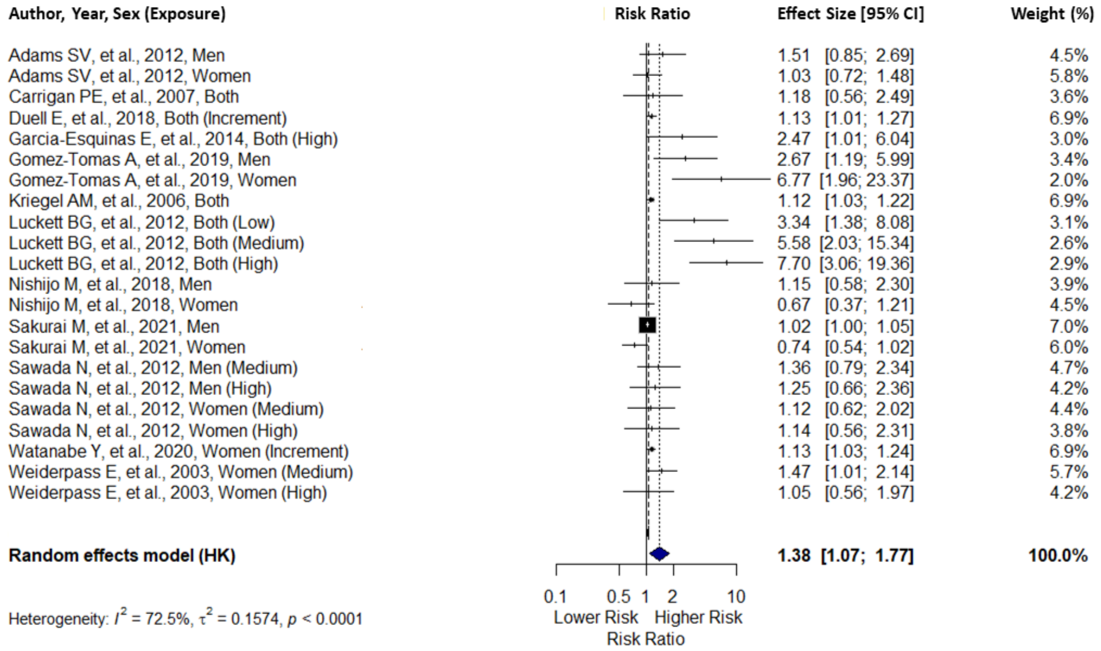

(B) Excluding top 10% highest-weighted studies

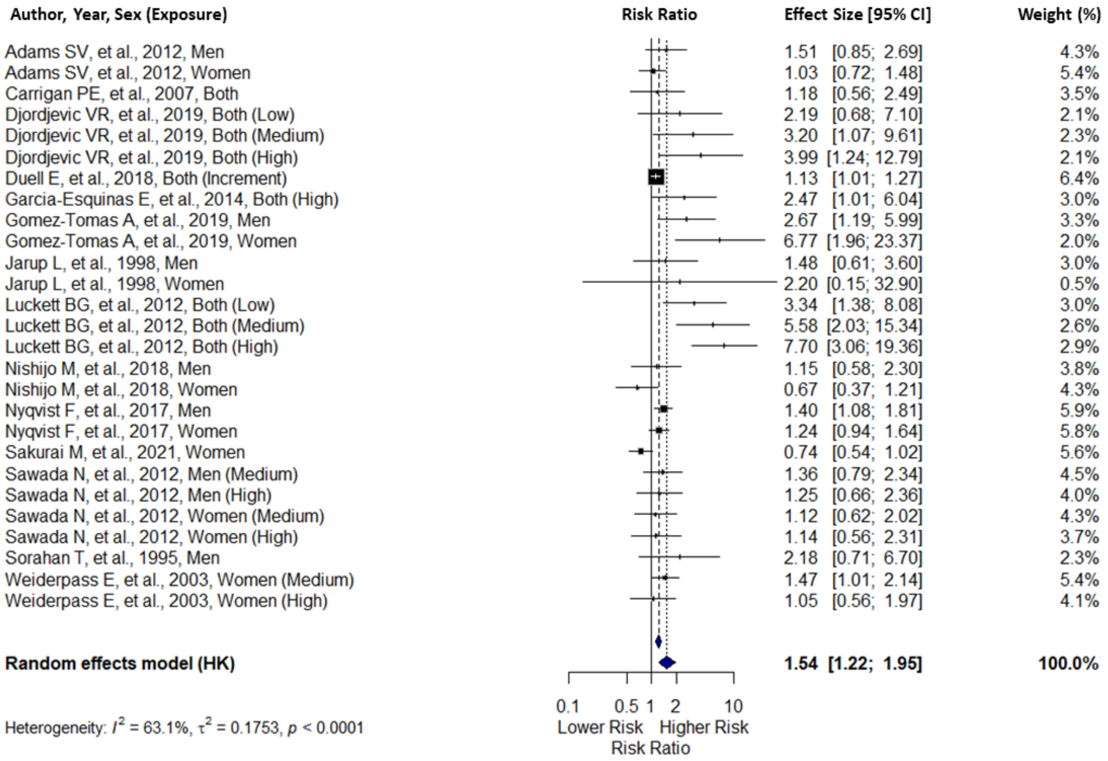

Figure S5. Sensitivity analyses of pooled relative risk for pancreatic cancer by alternative models: (a) by excluding studies at high risk of bias, categorized as Tier 2, (b) by excluding studies with top 10% of the highest weights in the main model.

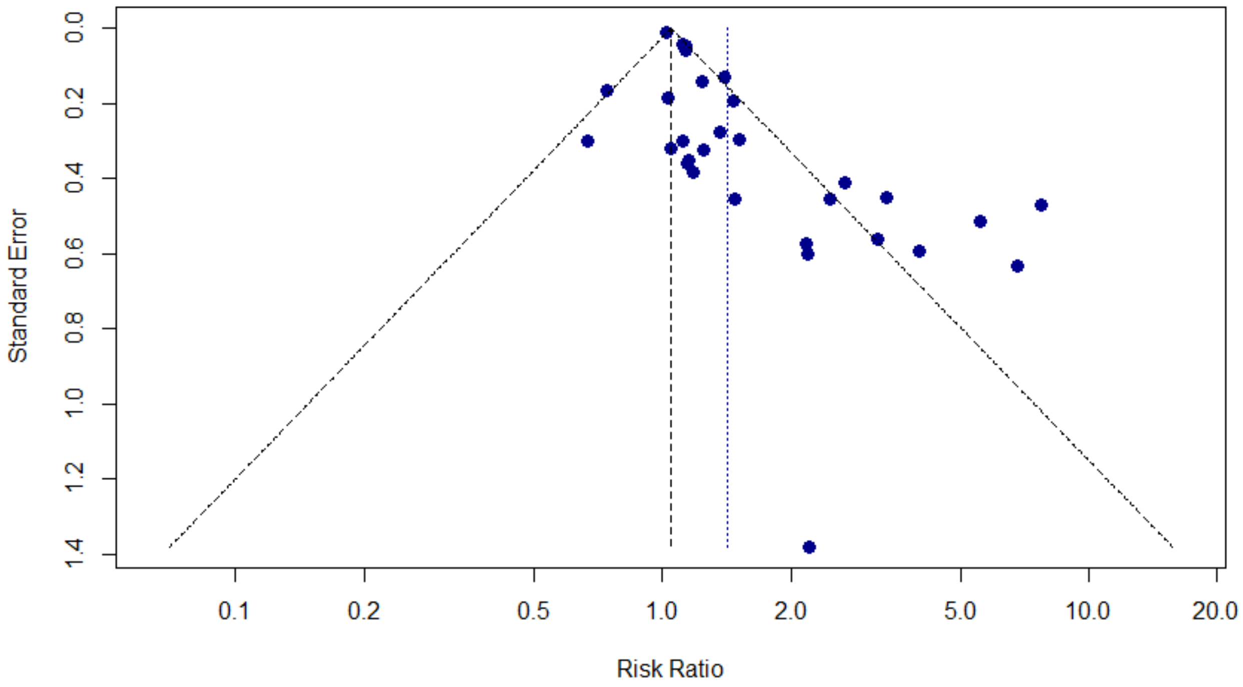

Figure S6. Funnel plot for the risk ratio from the articles included in the meta-analysis

(A) Main analysis

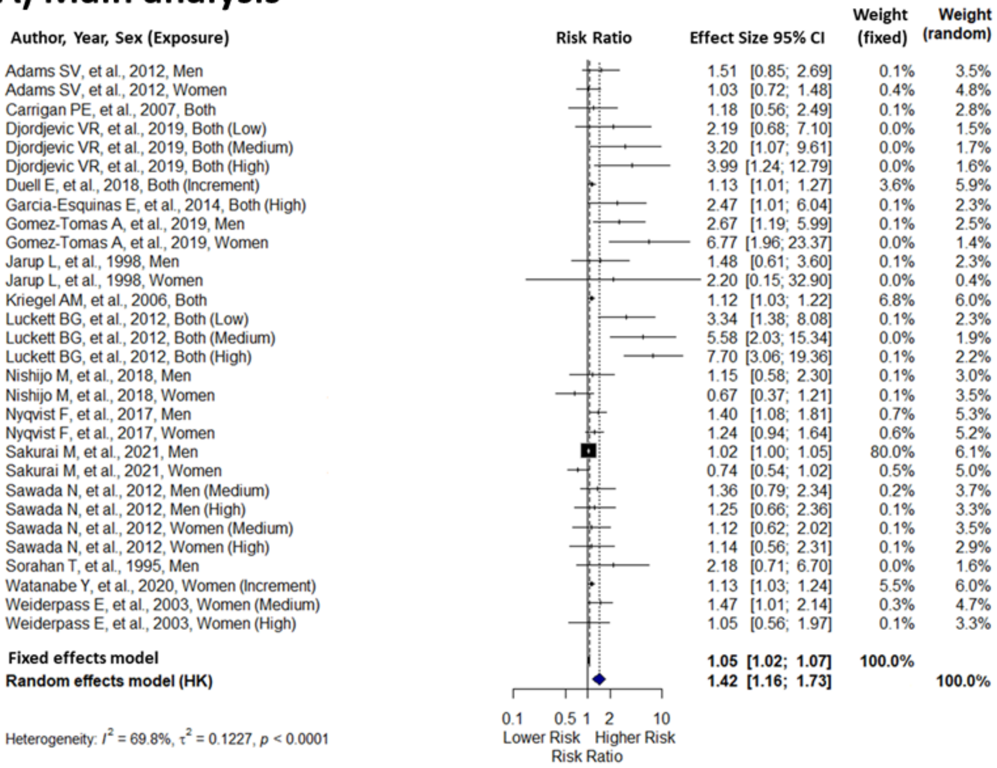

(B) Sensitivity analysis using one effect estimate per study

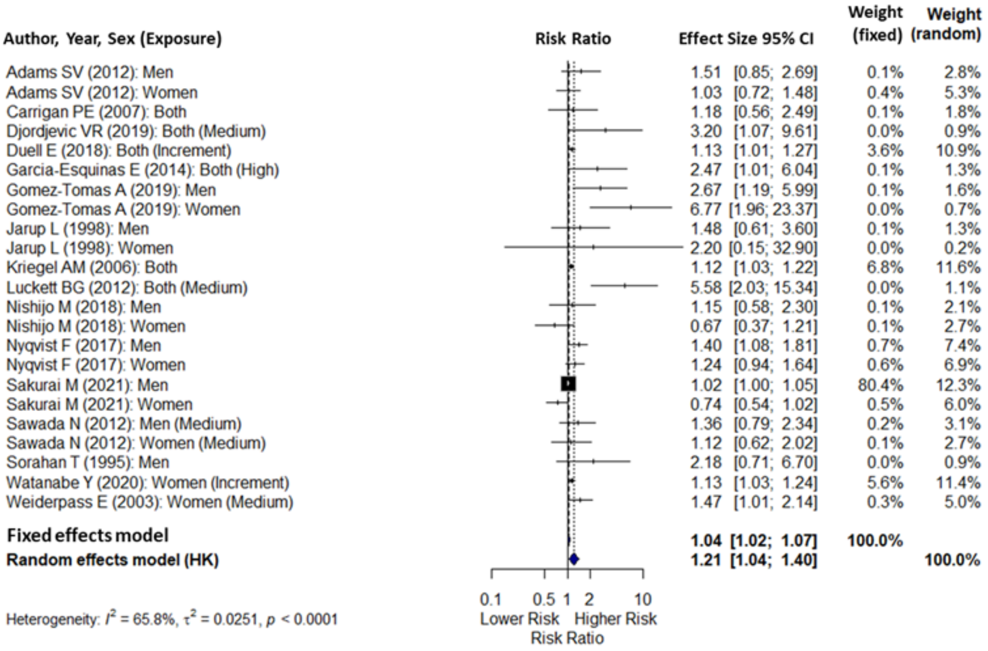

Figure S7. Forest plots comparing fixed-effect and random-effects estimates

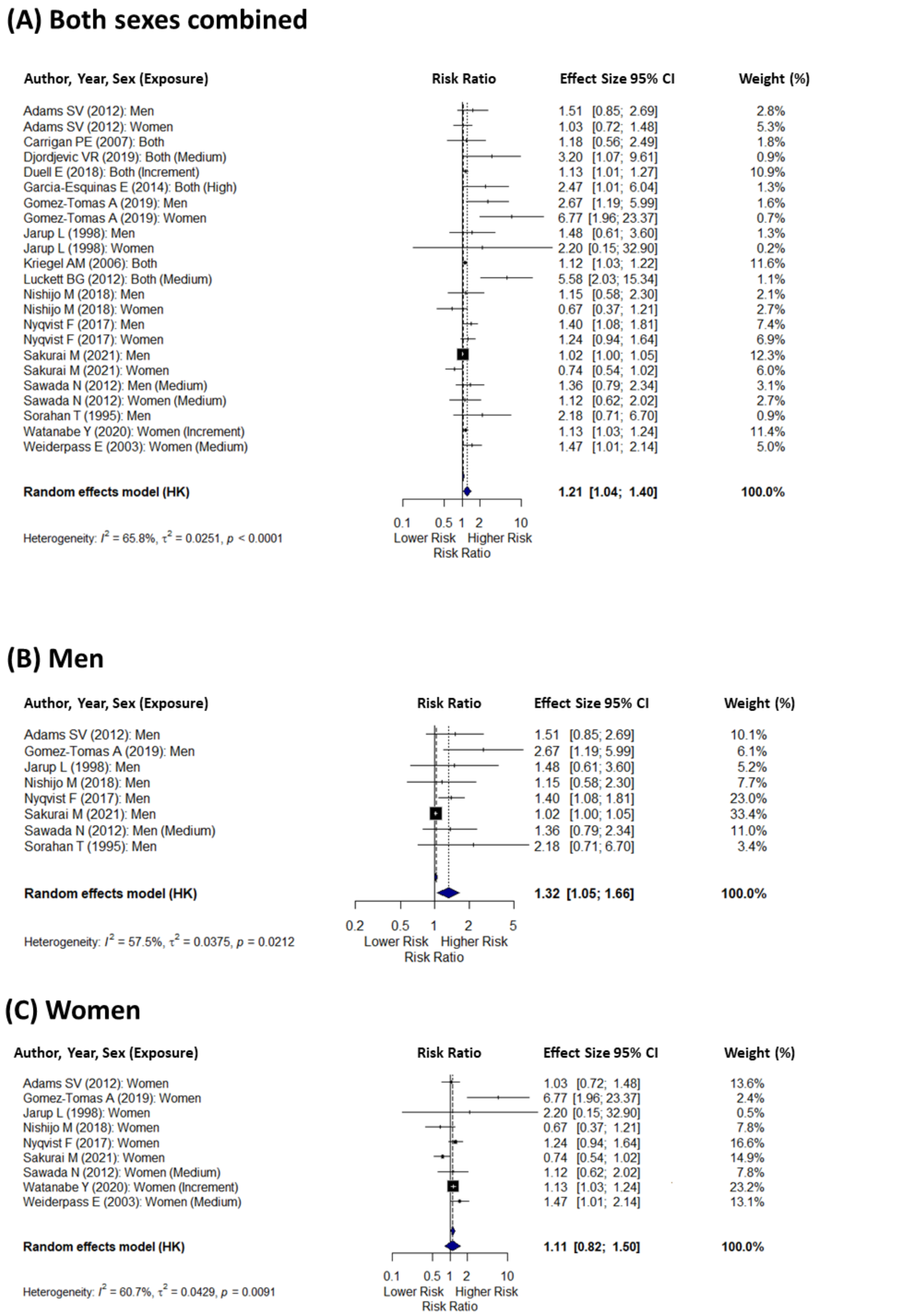

Figure S8. Forest plot of the pooled effect of cadmium exposure on pancreatic cancer risk, by sex (sensitivity analysis using one effect estimate per study)

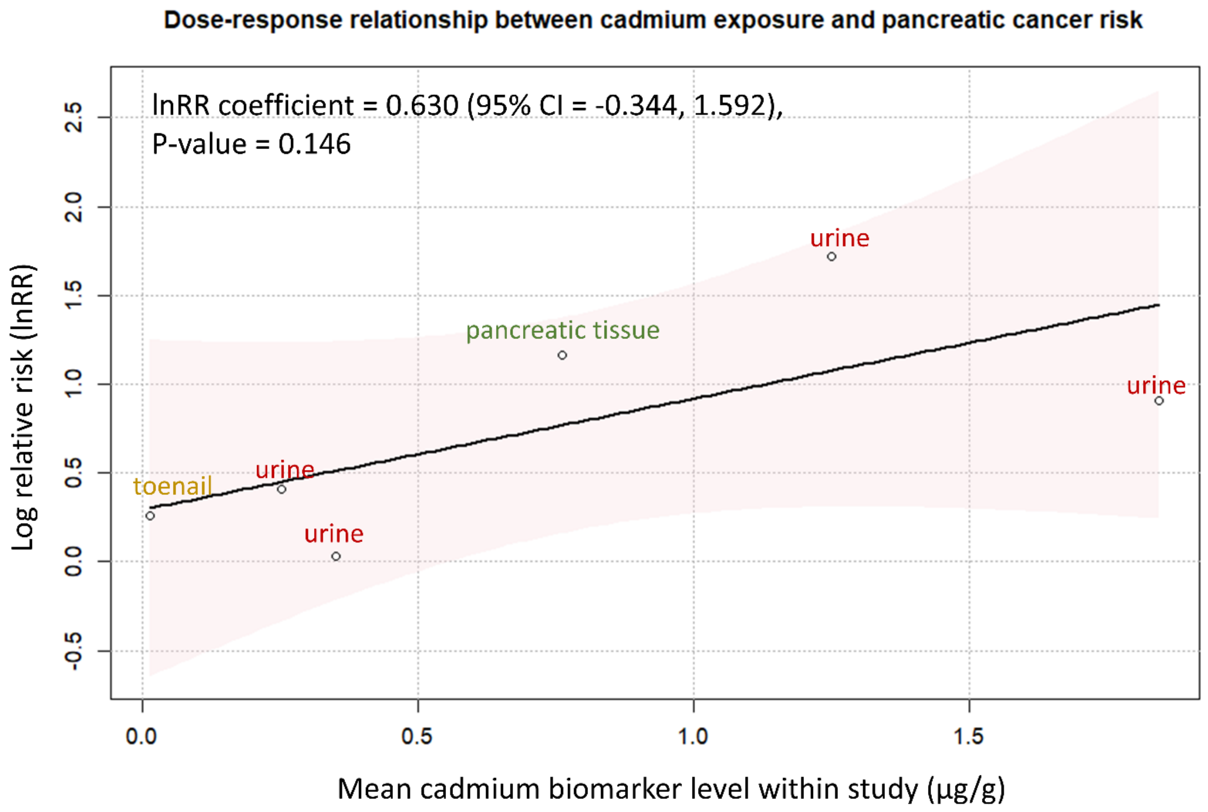

Figure S9. Meta-regression of the association between cadmium exposure and pancreatic cancer risk (sensitivity analysis using one effect estimate per study)

By region

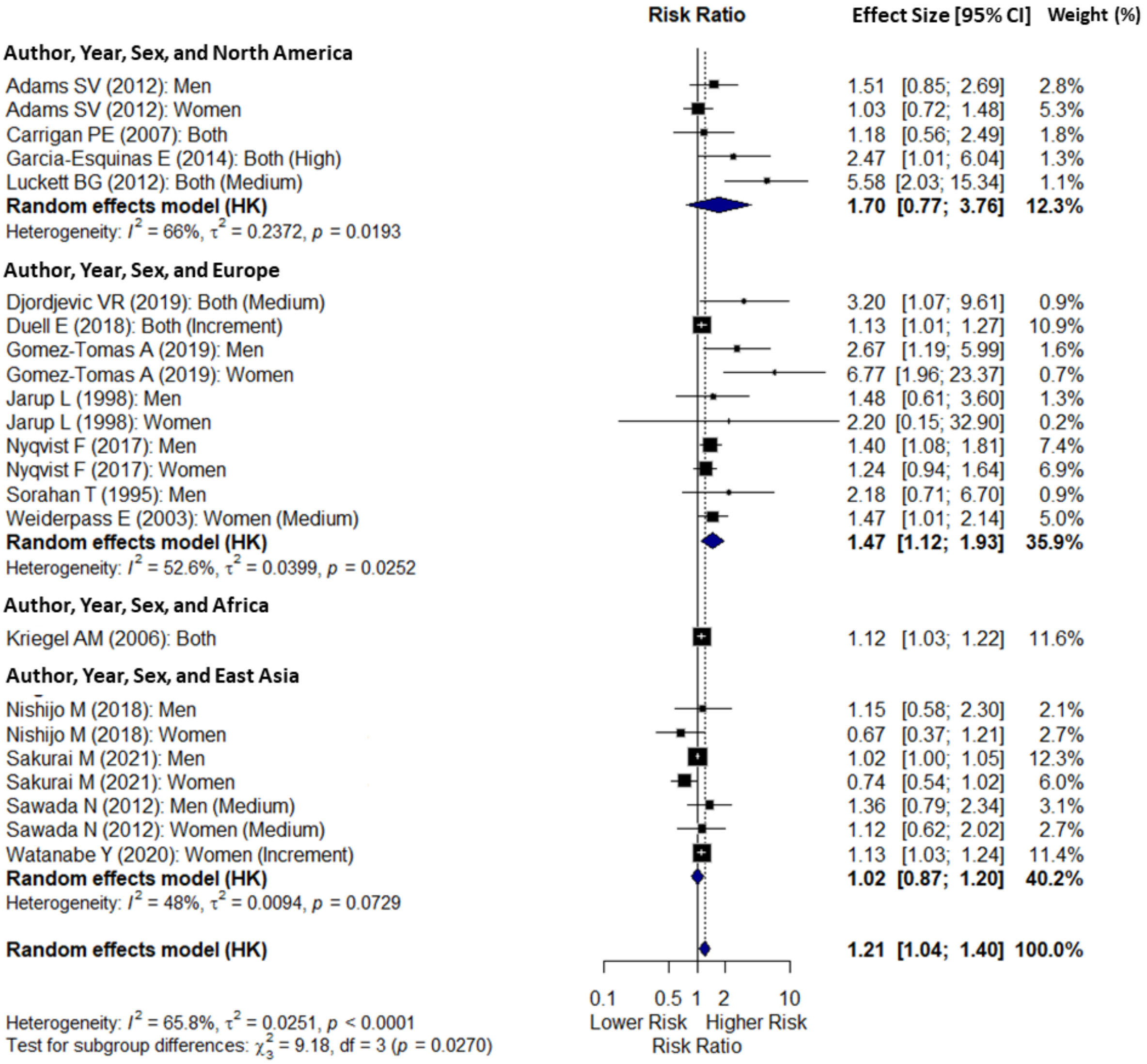

Figure S10. Subgroup analysis between cadmium exposure and pancreatic cancer risk, by regions (sensitivity analysis using one effect estimate per study)

By exposure status

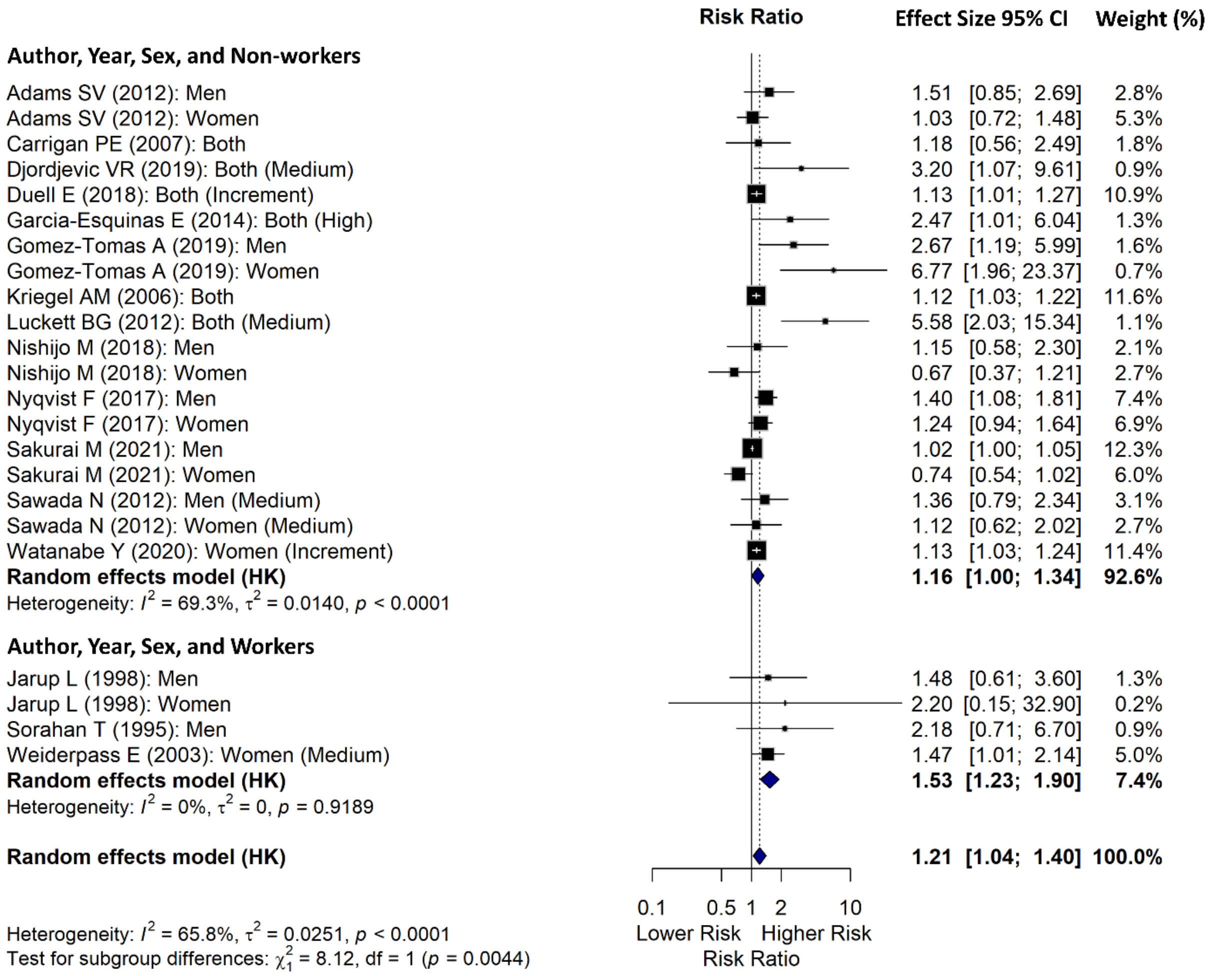

Figure S11. Subgroup analysis between cadmium exposure and pancreatic cancer risk, by exposure status (sensitivity analysis using one effect estimate per study)

(A) Urine and pancreatic tissue

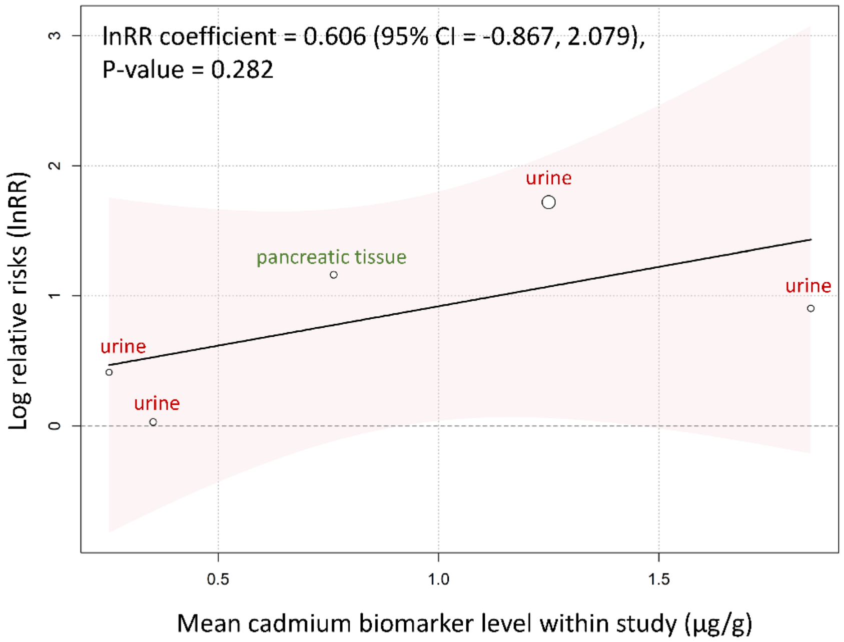

(B) Urine

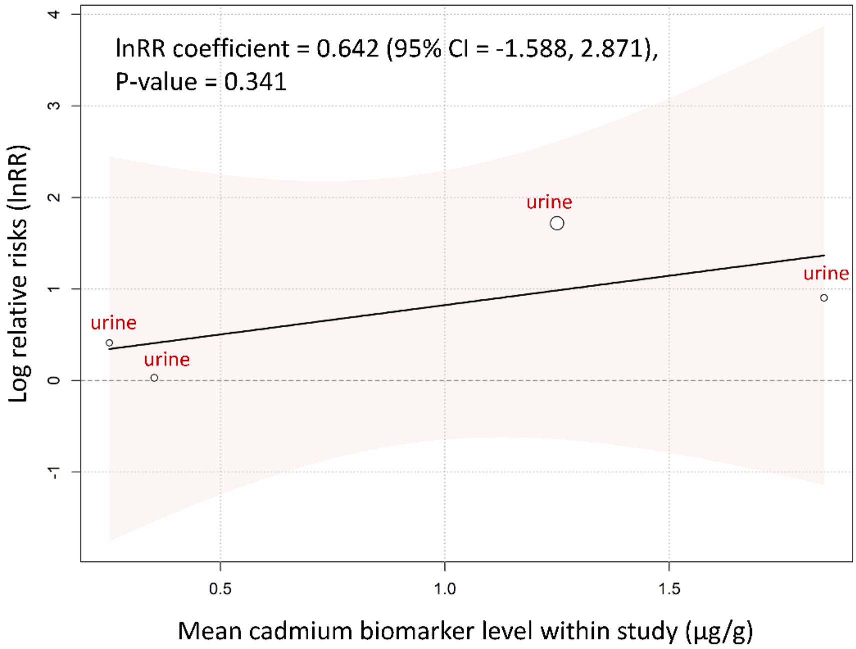

Figure S12. Meta-regression of the association between cadmium exposure and pancreatic cancer risk (sensitivity analysis using one effect estimate per study).

The plot shows the dose-response relationship between cadmium concentration (µg/g) and the natural logarithm of the relative risk (lnRR) for pancreatic cancer, based on a random-effects meta-regression model. The solid line represents the fitted trend, and the shaded area indicates the 95% confidence interval (CI). Each point represents a single study, with one effect estimate retained per study to address potential statistical dependency, defined by biomarker type as urine and pancreatic tissue. Cadmium concentrations reflect values reported within individual studies, and the lnRR represents the estimated change in risk per 1 µg/g increase in exposure.

(A) Excluding studies at high risk of bias (Tier 2 studies)

Author, Year, Sex (Exposure)	Effect Size [95% CI]	Weight (%)
Adams SV (2012): Men	1.51 [0.85; 2.69]	5.3%
Adams SV (2012): Women	1.03 [0.72; 1.48]	7.6%
Carrigan PE (2007): Both	1.18 [0.56; 2.49]	4.0%
Djordjevic VR (2019): Both (Medium)	3.20 [1.07; 9.61]	2.3%
Duell E (2018): Both (Increment)	1.13 [1.01; 1.27]	10.2%
Garcia-Esquinas E (2014): Both (High)	2.47 [1.01; 6.04]	3.1%
Gomez-Tomas A (2019): Men	2.67 [1.19; 5.99]	3.6%
Gomez-Tomas A (2019): Women	6.77 [1.96; 23.37]	1.9%
Jarup L (1998): Men	1.48 [0.61; 3.60]	3.2%
Jarup L (1998): Women	2.20 [0.15; 32.90]	0.5%
Luckett BG (2012): Both (Medium)	5.58 [2.03; 15.34]	2.6%
Nishijo M (2018): Men	1.15 [0.58; 2.30]	4.4%
Nishijo M (2018): Women	0.67 [0.37; 1.21]	5.2%
Nyqvist F (2017): Men	1.40 [1.08; 1.81]	8.8%
Nyqvist F (2017): Women	1.24 [0.94; 1.64]	8.6%
Sakurai M (2021): Women	0.74 [0.54; 1.02]	8.1%
Sawada N (2012): Men (Medium)	1.36 [0.79; 2.34]	5.6%
Sawada N (2012): Women (Medium)	1.12 [0.62; 2.02]	5.2%
Sorahan T (1995): Men	2.18 [0.71; 6.70]	2.2%
Weiderpass E (2003): Women (Medium)	1.47 [1.01; 2.14]	7.5%
**Random effects model (HK)**	**1.19 [1.10; 1.30]**	

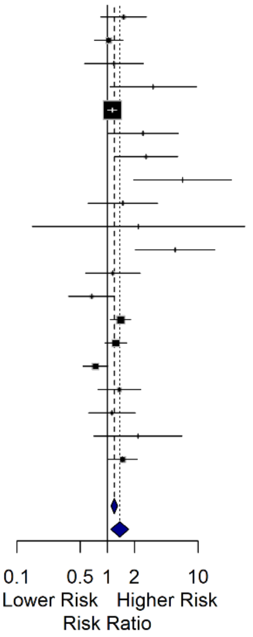

Heterogeneity: $I^2 = 57.5\%$, $\tau^2 = 0.0883$, $p = 0.0007$

(B) Excluding top 10% highest-weighted studies

Author, Year, Sex (Exposure)	Effect Size [95% CI]	Weight (%)
Adams SV (2012): Men	1.51 [0.85; 2.69]	1.6%
Adams SV (2012): Women	1.03 [0.72; 1.48]	3.7%
Carrigan PE (2007): Both	1.18 [0.56; 2.49]	1.0%
Duell E (2018): Both (Increment)	1.13 [1.01; 1.27]	15.8%
Garcia-Esquinas E (2014): Both (High)	2.47 [1.01; 6.04]	0.7%
Gomez-Tomas A (2019): Men	2.67 [1.19; 5.99]	0.8%
Gomez-Tomas A (2019): Women	6.77 [1.96; 23.37]	0.4%
Kriegel AM (2006): Both	1.12 [1.03; 1.22]	19.1%
Luckett BG (2012): Both (Medium)	5.58 [2.03; 15.34]	0.5%
Nishijo M (2018): Men	1.15 [0.58; 2.30]	1.1%
Nishijo M (2018): Women	0.67 [0.37; 1.21]	1.5%
Sakurai M (2021): Men	1.02 [1.00; 1.05]	24.6%
Sakurai M (2021): Women	0.74 [0.54; 1.02]	4.4%
Sawada N (2012): Men (Medium)	1.36 [0.79; 2.34]	1.8%
Sawada N (2012): Women (Medium)	1.12 [0.62; 2.02]	1.5%
Watanabe Y (2020): Women (Increment)	1.13 [1.03; 1.24]	18.2%
Weiderpass E (2003): Women (Medium)	1.47 [1.01; 2.14]	3.4%
**Random effects model (HK)**	**1.12 [0.99; 1.26]**	**100.0%**

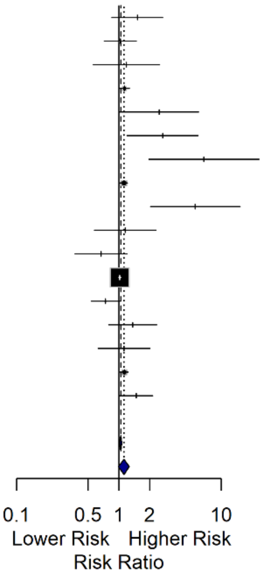

Heterogeneity: $I^2 = 68.8\%$, $\tau^2 = 0.0058$, $p < 0.0001$

Figure S13. Comparison of main analysis and sensitivity analysis using one effect estimate per study: (a) by excluding studies at high risk of bias, categorized as Tier 2, (b) by excluding studies with top 10% of the highest weights in the main model.

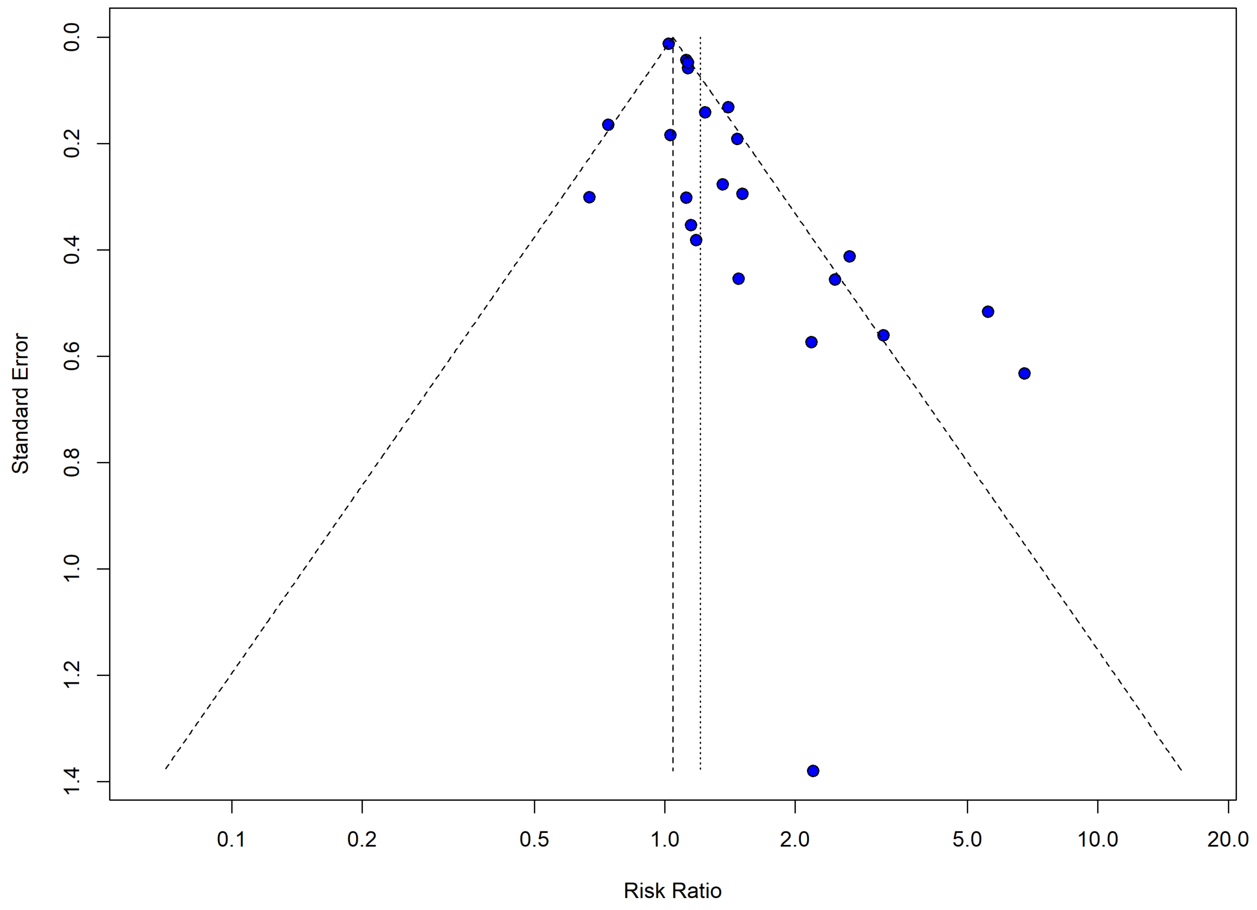

Figure S14. Funnel plot for the risk ratio from the articles included in the meta-analysis (sensitivity analysis using one effect estimate per study)

Table S4. Summary of the certainty of evidence

Number of studies	Study design	Risk of bias	Inconsistency	Indirectness	Imprecision	Publication bias	Upgrade	Effect	Certainty of evidence (GRADE)
**Meta-analysis: Pooled effect size**									
16	Observational studies	Serious	Serious	Not serious	Not serious	Serious	Dose-response gradient, residual bias towards the null	RR = 1.42 (95% CI = 1.16 to 1.73)	⊕⊕⊖⊖ **Low**
**Meta-analysis: Mean difference**									
7	Observational studies	Serious	Very serious	Not serious	Not serious	Serious		SMD = 1.26 (95% CI = -0.33 to 2.85)	⊕⊖⊖⊖ **Very Low**
**Meta-regression**									
5	Observational studies	Serious	Very serious	Not serious	Not serious	Serious	Dose-response gradient	lnRR coefficient = 0.610 (95% CI = 0.055 to 1.165)	⊕⊕⊖⊖ **Low**

Abbreviations: CI, confidence interval; GRADE, Grading of Recommendations Assessment, Development, and Evaluation; lnRR = natural log-transformed relative risk; RR = relative risk; SMD = standardized mean difference.

References

1. Office of Health Assessment and Translation (OHAT). Handbook for conducting a literature-based health assessment using OHAT approach for systematic review and evidence integration. National Toxicology Program, U.S. Department of Health and Human Services; 2019 [cited September 25, 2024]. Available from: https://ntp.niehs.nih.gov/sites/default/files/ntp/ohat/pubs/handbookmarch2019_508.pdf.

2. Turner HM, III, Bernard RM. Calculating and synthesizing effect sizes. Contemp Issues Commun Sci Disord. 2006;33:42-55.

3. Schünemann HJ, Higgins JPT, Vist GE, Glasziou P, Akl EA, Skoetz N, et al. Chapter 14: Completing 'Summary of findings' tables and grading the certainty of the evidence [last updated August 2023]. In: Higgins JPT, Thomas J, Chandler J, Cumpston M, Li T, Page MJ, Welch VA (editors). Cochrane Handbook for Systematic Reviews of Interventions version 6.5. Cochrane; 2024 [cited February 10, 2025]. Available from: https://training.cochrane.org/handbook/current/chapter-14.

4. Adams SV, Passarelli MN, Newcomb PA. Cadmium exposure and cancer mortality in the Third National Health and Nutrition Examination Survey cohort. Occup Environ Med. 2012;69(2):153-6.

5. Amaral AFS, Porta M, Silverman DT, Milne RL, Kogevinas M, Rothman N, et al. Pancreatic cancer risk and levels of trace elements. Gut. 2012;61(11):1583-8.

6. Baralic K, Javorac D, Maric D, Dukic-Cosic D, Bulat Z, Miljakovic EA, et al. Benchmark dose approach in investigating the relationship between blood metal levels and reproductive hormones: Data set from human study. Environ Int. 2022;165.

7. Wan X, Wang W, Liu J, Tong T. Estimating the sample mean and standard deviation from the sample size, median, range and/or interquartile range. BMC Medical Research Methodology. 2014;14(1):135.

8. Carrigan PE, Hentz JG, Gordon G, Morgan JL, Raimondo M, Anbar AD, et al. Distinctive heavy metal composition of pancreatic juice in patients with pancreatic carcinoma. Cancer Epidemiol Biomarkers Prev. 2007;16(12):2656-63.

9. Djordjevic VR, Wallace DR, Schweitzer A, Boricic N, Knezevic D, Matic S, et al. Environmental cadmium exposure and pancreatic cancer: Evidence from case control, animal and in vitro studies. Environ Int. 2019;128:353-61.

10. Duell E, Lujan-Barroso L, Outzen M, Raaschou-Nielsen O, Jenab M, Sund M, et al. Pre-diagnostic erythrocyte cadmium, selenium, and zinc levels and pancreatic cancer risk in Europe. Pancreatology. 2018;18(4):S55-S6.

11. Farzin L, Moassesi ME, Sajadi F, Ahmadi Faghih MA. Evaluation of trace elements in pancreatic cancer patients in Iran. Middle East J Cancer. 2013;4(2):79-86.

12. Forte G, Pisano A, Bocca B, Fenu G, Farace C, Etzi F, et al. Toxic metal and essential element

concentrations in the blood and tissues of pancreatic ductal adenocarcinoma patients. Toxics. 2024;12(1).

13. García-Esquinas E, Pollan M, Tellez-Plaza M, Francesconi KA, Goessler W, Guallar E, et al. Cadmium exposure and cancer mortality in a prospective cohort: The strong heart study. Environ Health Perspect. 2014;122(4):363-70.

14. Gómez-Tomás Á, Pumarega J, Alguacil J, Amaral AFS, Malats N, Pallarès N, et al. Concentrations of trace elements and KRAS mutations in pancreatic ductal adenocarcinoma. Environ Mol Mutagen. 2019;60(8):693-703.

15. Järup L, Bellander T, Hogstedt C, Spång G. Mortality and cancer incidence in Swedish battery workers exposed to cadmium and nickel. Occup Environ Med. 1998;55(11):755-9.

16. Kriegel AM, Soliman AS, Zhang Q, El-Ghawalby N. Serum cadmium levels in pancreatic cancer patients from the East Nile Delta region of Egypt. Environ Health Perspect. 2006;114(1):113-9.

17. Luckett BG, Su LJ, Rood JC, Fontham ETH. Cadmium exposure and pancreatic cancer in south Louisiana. J Environ Public Health. 2012;2012:180186-.

18. Nishijo M, Nakagawa H, Suwazono Y, Nogawa K, Sakurai M, Ishizaki M, et al. Cancer mortality in residents of the cadmium-polluted Jinzu River basin in Toyama, Japan. Toxics. 2018;6(2).

19. Nyqvist F, Helmfrid I, Augustsson A, Wingren G. Increased cancer incidence in the local population around metal-contaminated glassworks sites. J Occup Environ Med. 2017;59(5):E84-E90.

20. Sakurai M, Suwazono Y, Nishijo M, Nogawa K, Watanabe Y, Ishizaki M, et al. Relationship between urinary β(2) -microglobulin concentration and mortality in a cadmium-polluted area in Japan: A 35-year follow-up study. J Appl Toxicol. 2021;41(2):224-32.

21. Sawada N, Iwasaki M, Inoue M, Takachi R, Sasazuki S, Yamaji T, et al. Long-term dietary cadmium intake and cancer incidence. Epidemiology. 2012;23(3):368-76.

22. Sen G. Excretion of heavy metals from the biliary tract and its association with pancreato-biliary malignancy [Dissertation/Thesis]. U.K.: Newcastle University; 2015.

23. Sorahan T, Lister A, Gilthorpe MS, Harrington JM. Mortality of copper cadmium alloy workers with special reference to lung cancer and non-malignant diseases of the respiratory system, 1946-92. Occup Environ Med. 1995;52(12):804-12.

24. Watanabe Y, Nogawa K, Nishijo M, Sakurai M, Ishizaki M, Morikawa Y, et al. Relationship between cancer mortality and environmental cadmium exposure in the general Japanese population in cadmium non-polluted areas. Int J Hyg Environ Health. 2020;223(1):65-70.

25. Weiderpass E, Vainio H, Kauppinen T, Vasama-Neuvonen K, Partanen T, Pukkala E. Occupational exposures and gastrointestinal cancers among Finnish women. J Occup Environ Med. 2003;45(3):305-15.
